# HA-CCP: A Hybrid Algorithm for Solving Capacitated Clustering Problem

**DOI:** 10.1155/2022/6400318

**Published:** 2022-01-21

**Authors:** Yaoyao Liu, Ping Guo, Yi Zeng

**Affiliations:** ^1^College of Computer Science, Chongqing University, Chongqing 400044, China; ^2^Chongqing Key Laboratory of Software Theory and Technology, Chongqing 400044, China

## Abstract

The capacitated clustering problem (CCP) divides the vertices of the undirected graph into several disjoint clusters so that the sum of the node weights in each cluster meets the capacity limit while maximizing the sum of the weight of the edges between nodes in the same cluster. CCP is a typical NP-hard problem with a wide range of engineering applications. In recent years, heuristic algorithms represented by greedy random adaptive search program (GRASP) and variable neighborhood search (VNS) have achieved excellent results in solving CCP. To improve the efficiency and quality of the CCP solution, this study proposes a new hybrid algorithm HA-CCP. In HA-CCP, a feasible solution construction method is designed to adapt to the CCP with stricter upper and lower bound constraints and an adaptive local solution destruction and reconstruction method is designed to increase population diversity and improve convergence speed. Experiments on 90 instances of 4 types show that the best average solution obtained by HA-CCP on 58 instances is better than all comparison algorithms, indicating that HA-CCP has better solution stability. HA-CCP is also superior to all comparison algorithms in average solving efficiency.

## 1. Introduction

The capacitated clustering problem (CCP) divides the vertices of the undirected graph into several disjoint clusters so that the sum of the node weights in each cluster meets the capacity limit while maximizing the sum of the weight of the edges between nodes in the same cluster.

CCP is closely related to the graph partition problem (GPP) [1–5], where the goal is to find a partition of the vertex set in *k* classes while minimizing the number of cut edges and respecting a balance constraint between the classes. Moreover, the maximum diversity grouping problem (MDGP) [6–13] is used to partition the vertices of an edge-weighted and undirected complete graph into *m* groups such that the total weight of the groups is maximized subject to some group size constraints. Consequently, the objective of the handover minimization problem (HMP) in the mobile network [14] is to minimize the sum of weights of the edges with endpoints in different clusters. In addition, CCP also has essential applications in vehicle routing [15] and mail delivery [16].

Optimization problems in real life are becoming more complex, with large-scale, nonlinear, multi-constrain characteristics. In recent years, many intelligent optimization algorithms [17–22] have been proposed to solve some complex practical problems effectively. Since Mulvey and Beck [23] proposed the CCP model in 1984, much literature has been on CCP and related issues. The greedy random adaptive search program (GRASP) is an effective method to solve CCP. Deng and Bard [24] combined a GRASP with path relinking (PR) and proposed GRASP-PR with a postprocessing stage. Morán-Mirabal et al. [14] proposed three random heuristic methods to solve the handover minimization problem in mobile networks: a GRASP with path relinking for the generalized quadratic assignment problem (denoted by GQAP), a GRASP with evolutionary path relinking (denoted by GevPR-HMP), and a biased random-key genetic algorithm (BRKGA). Martínez-Gavara et al. [25] proposed the greedy random adaptive search program (GRASP), Tabu search method (TS), a hybrid method combining GRASP and TS (GRASP + TS), and Tabu search with strategic oscillation (TS_SO). Martínez-Gavara et al. [26] proposed three random heuristic algorithms: greedy random adaptive search process (GRASP2-1), iterative greedy (IG), and their hybrid IG-GRASP.

Algorithms based on variable neighborhood search (VNS) [27, 28] are another method of solving CCP. Lai and Hao [29] proposed an iterative variable neighborhood search (IVNS) algorithm. Brimberg et al. [30] proposed the general VNS (GVNS) and the skewed general VNS (SGVNS). Lai *X* et al. [31] proposed the neighborhood decomposition-driven variable neighborhood search (NDVNS) for capacitated clustering. In addition, a Tabu search designed specifically for CCP includes the TS (denoted as FITS) and the memetic algorithm (MA) proposed in the literature [32].

CCP is an NP problem. By imposing upper and lower limits on the size of clusters, CCP becomes a typical constrained clustering problem. The complexity of CCP is related to the scale of the problem and the upper and lower limits of its capacity. Large-scale CCPs require more calculation time to obtain a good solution than those of small and medium CCPs. In particular, it is challenging to find feasible solutions for CCPs with strict upper or lower limits. Literature [26] introduced a hybrid method called IG-GRASP to solve CCP. On the one hand, IG-GRASP initializes a new solution and restarts the search when it falls into the local optimum and fails to break out the local optimum after a preset number of searches. This process discards the results of the previous stage search. As mentioned in [26], it is more efficient to construct a link solution of the current solution than to construct a new solution. On the other hand, as described in Section 3.3, the destruction strength of the current solution by the IG-GRASP destructive method depends on the number of clusters and nodes of the instance. When applying IG-GRASP to solve large-scale instances, the destructive method may excessively destroy the current solution, which is not conducive to the final convergence of the algorithm to a better solution. In addition, the construction method of IG-GRASP is difficult to successfully construct feasible initial solutions for some instances with strict upper and lower limits of capacity. Recently, algorithms based on VNS have been proposed to solve CCP, including IVNS, GVNS, and SGVNS. The experimental results in the literature [32] show that SGVNS has the best performance. We believe this is mainly due to the extension of the search to other promising areas of the solution space by adding skewed acceptance criteria. The algorithms based on VNS all use a random shaking process to introduce diversity to search. To improve the efficiency and quality of CCP solutions, this study proposes a new hybrid algorithm HA-CCP, which intelligently combines IG-GRASP and SGVNS algorithms. HA-CCP follows the framework of SGVNS and uses partial destruction and reconstruction strategies to introduce diversity to search. In addition, we also designed a new initial solution constructive method and partial destructive method to avoid the above limitations in IG-GRASP. The main contributions of this study include the following:

1. A solution construction method CM2 is designed. Even if the upper and lower boundaries of the cluster are very tight, CM2 can successfully build a feasible solution. It can construct feasible solutions in a wider range of CCP solutions to make the algorithm run correctly and perform better than the random construction method [29, 31, 32].
2. A destructive method DM3 is designed to destruct partial solutions. It adaptively destructs the solution according to the number of clusters and nodes of the instance while limiting the maximum number of deleted nodes in large-scale instances. In large-scale instances, the performance of DM3 is significantly better than DM2 [26].
3. A hybrid algorithm HA-CCP is proposed to solve CCP. The HA-CCP follows the framework of SGVNS, uses the partial destruction and reconstruction strategy to shake the current solution, uses the proposed solution constructor to construct the initial solution, reconstructs the partial solution, and uses the proposed destruction method to destroy the partial solution. Compared with the existing VNS-based CCP algorithm [29–31], HA-CCP uses a non-fully random method to shake the current solution to improve the efficiency of the algorithm and the quality of the solution.

The experimental results on 90 benchmark test instances show that HA-CCP is superior to all comparison algorithms except NDVNS (because NDVNS [31] does not provide calculation time) in terms of average solving efficiency. In terms of solution quality, HA-CCP obtained 57 best average results and 30 best results, indicating that HA-CCP outperformed all comparison algorithms in average results. However, the solution quality of HA-CCP on MDG-a instances is inferior to NDVNS.

The rest of the study is organized as follows. In Section 2, the definition of the CCP and the latest heuristics for CCP are introduced. Then, the overall framework of HA-CCP and the proposed construction method CM2 and destruction method DM3 are introduced in Section 3.  Section 4 gives the experimental results and time-to-target analysis on benchmark instances.  Section 5 analyzes the contribution of the critical algorithmic component to the performance of the proposed algorithms. Finally, conclusions and future work are provided in Section 6.

## 2. Background and Literature Review

This section introduces the concept of the CCP and the state-of-the-art CCP algorithms in the literature. Some of these algorithms are also used for analysis and comparison in Section 4.

### 2.1. Basic Concepts

For undirected weighted graphs, *G* =(*V*,  *E*), where *V* = {*v*_1_,  *v*_2_,   …,  *v*_*n*_} is a set of *n* nodes and *E* is the set of edges. *w*_*i*_  ≥  0 is the weight of the node *v*_*i*_  ∈  *V*.  *c*_*ij*_ is the weight of the edge. *V* is divided into *p* clusters *V*_*k*_ ⊂  *V*, and *V*_*i*_∩*V*_*j*_=∅(*i*, *j* ∈ {1,2,…, *p*} and *i* ≠ *j*). If *v*_*i*_ ∈  *V*_*g*_, let the binary variable *X*_*ig*_ = 1; otherwise, *X*_*ig*_ = 0,  *i*= 1,  2,  ...,  *n*;  *g* = 1,  2,  ...,  *p*. The CCP based on *G* can be expressed as an optimization problem [29, 32]:

(1) $$\begin{array}{c} \text{maximize}\sum_{g=1}^{p}\sum_{i=1}^{n-1}\sum_{j=i+1}^{n}c_{ij}X_{ig}X_{jg}, \end{array}$$

(2) $$\begin{array}{c} \text{subject to }\sum_{g=1}^{p}X_{ig}=1,\;\forall v_{i}\in V, \end{array}$$

(3) $$\begin{array}{c} \begin{array}{c} L_{g}\leq \sum_{i=1}^{n}w_{i}X_{ig}\leq U_{g}\;,\;\forall g\in \left\{1,\ldots ,p\right\} \end{array}, \end{array}$$

(4) $$\begin{array}{c} \begin{array}{c} X_{ig}\in \left\{0,1\right\},\;\forall v_{i}\in V,\;g\in \left\{1,\ldots .p\right\} \end{array}, \end{array}$$

Here, equation (2) requires that each node be allocated and only allocated to one cluster, and equation (3) requires that the sum of the node weight of each cluster is not less than the lower limit of capacity *L*_*g*_ and does not exceed the upper limit of capacity *U*_*g*_. The subset of the solution *x* is called the partial solution of *x*.

### 2.2. IG-GRASP for CCP

IG-GRASP [26] is the latest algorithm for solving CCP based on GRASP, as shown in Algorithm 1. It first constructs an initial solution with the construction method CM (line 6) and then improves the initial solution with the local search method IM2-1 (line 7). Subsequently, the algorithm repeatedly applies the destructive method DM2 to destruct the solution (line 9), the CM to reconstruct the solution (line 10), and the IM2-1 for local search (line 11). If several attempts cannot improve the solution (lines 8–18), the algorithm constructs a new solution (line 6) and starts again.

In Algorithm 1, CM (line 6) is used to construct a feasible solution. It starts by seeding the *p* clusters *V*_1_, *V*_2_,…, *V*_*p*_ with *p* randomly selected nodes. Then, the clusters are explored in lexicographical order assigning nodes until all of them satisfy the lower bound constraint. To do so, the candidate list VCL is formed with all the unassigned nodes and the value *I*(*v*_*i*_,  *k*)=∑_*j*∈*V*_*k*__*c*_*ij*_ is calculated for all pairs (*i*,  *k*) of nodes and clusters. RVCL_*k*_, the restricted candidate list of nodes for cluster *k*, can be formed as follows:

(5) $$\begin{array}{c} \begin{array}{c} {\text{RVCL}}_{k}=\left\{v_{i}\in {\text{VCL}}_{k}:I\left(v_{i},k\right)\geq \alpha I_{\max}\left(v_{i},k\right)\right\} \end{array}, \end{array}$$

where *I*_max_(*k*)=max *I*(*v*_*i*_, *k*), *α* ∈ [0,1] is a parameter used to balance randomness and greed. Finally, CM randomly selects a node in RVCL_*k*_ and assigns to cluster *k*. The nodes are allocated in this way until cluster *k* satisfies the lower bound of capacity. Then, all clusters are processed in this way until all clusters meet the lower limit of capacity. In the next step, CM assigns all unallocated nodes to these clusters so that each cluster meets the capacity limit constraint. In particular, CM constructs the candidate list CL by equation (6), builds RCL by equation (7), selects one pair (*i*, *k*) at random, and assigns node *i* to cluster *k*. CM stops when all the nodes have been assigned in this way.

(6) $$\begin{array}{c} \begin{array}{c} \text{CL}=\left\{\left(i,k\right):1\leq k\leq p,\;W_{k}+w_{i}\leq U_{k}\right\}\mathrm{where}\;W_{k}=\underset{j\in k}{\sum }w_{j} \end{array}, \end{array}$$

(7) $$\begin{array}{c} RCL=\left\{\left(i,k\right)\in CL:I\left(v_{i},k\right)\geq \alpha I_{\max}\left(v,k\right)\right\}. \end{array}$$

DM2 (Algorithm 1, line 9) is used to destruct part of the solution. It aims to remove some nodes from the cluster so that those nodes can be assigned to a different cluster to increase the value of the objective function. DM2 first constructs a deleted candidate list DCL_*k*_ for the cluster *k* (*k* = 1,  2,  ...,  *p*) as follows:

(8) $$\begin{array}{c} \begin{array}{c} {\text{DCL}}_{k}=\left\{v_{i}\in V_{k}:C_{R}\left(v_{i},V_{k}\right)\leq \gamma_{k}\right\} \end{array}, \end{array}$$

where  *C*_*R*_(*v*_*i*_, *V*_*k*_)=*C*(*v*_*i*_, *V*_*k*_)/*C*(*v*_*i*_) is the relative contribution of node *v*_*i*_ to cluster *k*, *C*(*v*_*i*_, *V*_*k*_)= ∑_*v*_*j*_∈*V*_*k*__*c*_*ij*_ is the contribution of node *v*_*i*_ to the objective function value in cluster *k*, and *C*(*v*_*i*_)=∑_*v*_*j*_∈*V*_*c*_*ij*_  is the potential contribution of the node *v*_*i*_ to the objective function value, *γ*_*k*_=*δ* min_*v*_*i*_∈*V*_*k*__*C*_*R*_(*v*_*i*_, *V*_*k*_)+(1 − *δ*)max_*v*_*i*_∈*V*_*k*__*C*_*R*_(*v*_*i*_, *V*_*k*_) is a threshold, and *δ* ∈ [0,1] is a parameter to control the size of the deleted candidate list. The DM2 method deletes the percentage *β* elements from DCL_*k*_ of each cluster *k*. So, the actual number of nodes removed from cluster *k* is max (1,  ⌊*β*|DCL_*k*_|⌋), where |DCL_*k*_| is the length of the list DCL_*k*_.

### 2.3. VNS Algorithms for CCP

The VNS algorithm is a classic local search algorithm for combinatorial and global optimization problems (see [33–36]). It first constructs a feasible initial solution and then iteratively applies a shaking and local search to find the global optimal solution. Lai and Hao [29] followed the general VNS framework and proposed iterative variable neighborhood search (IVNS) for CCP. IVNS constructs the initial solution with a random construction procedure, shakes the current solution with a random shaking procedure, and finds the local optimal solution with the extended variable neighborhood descent (EVND).

Brimberg et al. [30] proposed two heuristic algorithms based on VNS to solve CCP. The first is to combine variable neighborhood descent (VND) with general VNS to obtain the general variable neighborhood search (GVNS). The second is to combine VND with skewed VNS to obtain skewed general variable neighborhood search (SGVNS). SGVNS expands the search range to other promising areas of the solution space by a skew operation. The acceptance criteria for skewed moves in SGVNS are described as follows:

(9) $$\begin{array}{c} \frac{f\left(x^{\prime \prime }\right)}{f\left(x^{b}\right)}+\varepsilon \;d\left(x^{\prime \prime },x^{b}\right)>1\text{ and }\frac{f\left(x^{\prime \prime }\right)}{f\left(x\right)}+\varepsilon \;d\left(x^{\prime \prime },x\right)>1, \end{array}$$

where *x*^*b*^ is the best solution, *x* is the current solution, and *x*^″^ is the new solution obtained after one application of shaking and local search. *ε* is a parameter, and function *d* denotes a measure of distance between two solutions, according to [30], and *d*(*x*^″^, *x*^*b*^) can be calculated as follows:

(10) $$\begin{array}{c} d\left(x^{\prime \prime },x^{b}\right)=\frac{\left|\left\{\left(i,j\right):\left(\left(g_{x^{\prime \prime },i}=g_{x\prime \prime ,j}\right)\wedge \left(g_{x^{b},i}\neq g_{x^{b},j}\right)\right)\vee \left(\left(g_{x^{b},i}=g_{x^{b},j}\right)\wedge \left(g_{x^{\prime \prime },i}\neq g_{x^{\prime \prime },j}\right)\right)\right\}\right|}{\left|\left\{\left(i,j\right):\left(g_{x^{\prime \prime },i}=g_{x^{\prime \prime },j}\right)\wedge \left(g_{x^{b},i}=g_{x^{b},j}\right)\right\}\right|}, \end{array}$$

where 1 ≤ *i* < j ≤ *n* and *g*_*x*^″^,*i*_ is the label of the cluster where the node *i* is located in the solution *x*^″^.

### 2.4. Tabu Search

FITS [32] is a Tabu search approach to solve CCP. It starts from generating feasible solutions and then enters the searching stage until the running deadline is reached. The searching stage alternates between a feasible local search phase (FLS for short) and an infeasible local search phase (InfLS for short). If FLS is trapped in a deep local optimum, FITS will switch to InfLS to guide the search towards new search regions. It alternately explores the feasible and infeasible solution space to avoid falling into the local optimum easily.

## 3. A Hybrid Algorithm for CCP: HA-CCP

In this section, we propose a hybrid algorithm HA-CCP to solve CCP. HA-CCP uses a process based on the combination of greediness and randomization to construct the initial solution, uses the VND method to perform a local search to find the local optimal solution, and uses the destruction and reconstruction partial solution to shake the current solution. In addition, an acceptance criterion for skewed moves was added to enable HA-CCP to extend the search range to other promising areas of the solution space, thereby reducing repeated searches for some areas of the solution space and increasing the diversity of the search. For ease of description, in the rest of this study, *x*^*b*^ represents the best solution obtained so far, *x* represents the current solution, and *x*^″^ represents the new solution obtained after one application of destruction, reconstruction, and local search of *x*.

### 3.1. HA-CCP Framework

The overall framework of HA-CCP is shown in Algorithm 2. It constructs an initial solution *x* with CM2 (Algorithm 2 line 2; see Section 3.2), and if *x* is not a feasible solution (Algorithm 2 line 3), an empty solution is returned (Algorithm 2 line 4); otherwise, *x* is improved with VND [30] (Algorithm 2 line 5) and the improved solution is copied as the current best solution *x*^*b*^ (Algorithm 2 line 6). Then, it enters the iterative process to optimize the solution until the loop condition is no longer satisfied. Each iteration consists of four stages: (1) the current solution is partially destructed (Algorithm 2 line 8; see Section 3.3); (2) the destroyed solution is reconstructed (Algorithm 2 line 9); if *x*″is not a feasible solution (Algorithm 2 line 10), *x*″ is updated with *x* (Algorithm 2 line 11); (3) VND is used to perform a local search on the reconstructed solution to obtain a new solution *x*″ (Algorithm 2 line 13); and (4) if the new solution *x*″ is better than the best solution *x*^*b*^, the current solution *x* and the best solution *x*^*b*^ are updated with the new solution *x*″; otherwise, whether to accept the new solution *x*″ as the current solution *x* according to acceptance criteria is determined. If the acceptance criteria were satisfied, the new solution *x*″ is accepted as the current solution *x.* Otherwise, the current solution *x* is not updated (Algorithm 2 lines 14–21). Because the three processes of partial destruction, reconstruction, and local search have a certain degree of randomness, each iteration will produce a different new solution.

Compared with Algorithm 1 (IG-GRASP), Algorithm 2 (HA-CCP) has the following differences:

1. *Local Search.* In Algorithm 1, IM2-1 has to be used (Algorithm 1, line 7) to improve the solution, which searches in neighborhood N4(*x*) [25], but Algorithm 2 applies the VND [30] based on 3 neighborhoods (Algorithm 2, line 5)
2. *Partial Destruction Solution*. Algorithm 1 partially destructs the solution with DM2 (Algorithm 1, line 9), but Algorithm 2 adopts DM3 (Algorithm 2, line 8)
3. *Acceptance Criteria*. Algorithm 1 adopts the “always replace” acceptance criterion (Algorithm 1, lines 12–17), but Algorithm 2 adopts the “conditional acceptance” criterion (Algorithm 2, lines 18–20)
4. *Jump out of the Local Optimum*. Algorithm 1 jumps out of the local optimization after the preset number of iterations without any improvement (Algorithm 1, Line 8), but Algorithm 2 appropriately accepts the suboptimal solution as the current solution to moving the search to other areas of the solution space (Algorithm 2, line 19)
5. Algorithm 1 has one possible starting point for a new round of search: the current solution *x* (Algorithm 1, lines 12–17), but Algorithm 2 has two: the current solution *x* and the new solution *x*″(Algorithm 2, lines 14–21). Therefore, Algorithm 2 has a larger optimization search space than Algorithm 1

Compared with the SGNVS algorithm, Algorithm 2 (HA-CCP) has the following differences:

1. In terms of the construction of the initial solution, HA-CCP uses a combination of randomization and greediness to construct the initial solution, and SGNVS uses a completely random construction method to construct the initial solution.
2. In terms of introducing diversity for search, HA-CCP adopts the strategy of destructing and reconstructing the current solution, and the VNS-based SGVNS adopts a completely random shake process.
3. In terms of perturbation strength, HA-CCP controls the number of the deleted nodes of the destructive method according to the number of nodes, clusters, and the parameter *d*_max of the current instance, while SGNVS adjusts the perturbation strength according to the solution obtained in the search process.

### 3.2. Construction Method: CM2

To construct a feasible solution, we propose CM2 in Algorithm 3. CM2 includes two steps. The first step (Algorithm 3, lines 4–16) is as follows: assigning nodes to each cluster until all clusters meet the lower limit of capacity. For cluster *p*(1 ≤ *k* ≤ *p*), CM2 first constructs a candidate list VCL_*k*_ with all the unallocated nodes (Algorithm 3, line 6). If the VCL_*k*_ is not empty (Algorithm 3, lines 7–10), the increment VCL_*k*_ of the objective function value is calculated and the restricted candidate list RVCL_*k*_ isconstructed by equation (5). Then, a node in RVCL_*k*_ is randomly selected and assigned to cluster *k*. If VCL is empty (Algorithm 3, lines 11–14), it indicates that all nodes have been allocated, but the cluster in solution *x* does not meet the lower limit of capacity. In this case, we apply Algorithm 4 to fix *x* into a feasible solution.

The second step (Algorithm 3, lines 18–29) is as follows: assigning the remaining unallocated nodes to suitable clusters. It first constructs the candidate list CL by equation (6). If CL is not empty (Algorithm 3, lines 20–23), the restricted candidate list RCL isconstructed by equation (7). Then, a pair of elements (*v*,  *k*) in RCL is randomly selected and *v* is assigned to cluster *k*. All unallocated nodes are allocated in this way. If the CL is empty (Algorithm 3, lines 24–27), it indicates that there is an unallocated node *v*, and allocating it to any cluster will cause the cluster capacity to exceed the upper limit. For this case, we apply the LWA (Algorithm 5) for processing. CM2 performs the solution construction process up to 10 times (Algorithm 3, lines 1–3). If the feasible solution is successfully constructed, it will jump out of the loop and return to the feasible solution (Algorithm 3, lines 30–34).

Algorithm 4 describes the repair method. For the cluster *g* that does not meet the lower limit of capacity, we first construct a feasible move-in node candidate list  MCL_*g*_ (Algorithm 4, line 3) by equation (11). If  MCL_*g*_ is empty (Algorithm 4, line 4), the infeasible solution *x* (Algorithm 4, line 5) is returned; otherwise, the restricted candidate list RMCL_*k*_ is constructed in a similar way to the restricted candidate list RVCL_*k*_ (Algorithm 4, line 6). Then, a node in RMCL_*k*_ is randomly selected and it is moved to the cluster *g*(Algorithm 4, lines 7–8). The node is moved in this way until all clusters meet the lower capacity constraint, and the repair ends.

(11) $$\begin{array}{c} \begin{array}{c} {\text{MCL}}_{g}=\left\{v_{i}:1\leq i\leq n,\;v_{i}\in V_{s},\;1\leq s\leq p,\;s\neq g,\;W_{s}-w_{i}\geq L_{s},W_{g}+w_{i}\leq U_{g}\right\}. \end{array} \end{array}$$

LWA is shown in Algorithm 5. It first selects a node *v* with the largest weight among the unallocated nodes (Algorithm 5, line 2). Then, as in equation (6), a feasible allocation candidate list CCL is formed just for node *v* (Algorithm 5, line 3). Second, if CCL is empty (Algorithm 5, line 4), the infeasible solution *x* (Algorithm 5, line 5) is returned. Otherwise, the restricted candidate list RCCL is constructed by equation (7) (Algorithm 5, line 6). Finally, a pair (*v*, *g*) is selected randomly from RCCL, and the node *v* is assigned to cluster *g* (Algorithm 5, lines 7–9). The remaining unallocated nodes are allocated according to the above way.

### 3.3. Destruction Method: DM3

To destruct partial solution, [26] proposed DM2. MD2 deletes at least one node for each cluster. For large instances, as the number of nodes and clusters increases, the number of deleted nodes increases for each cluster. On the one hand, it increases the time cost, and on the other hand, it is difficult to converge to a high-quality solution due to too many deleted nodes. To limit the number of deleted nodes, we propose a new destruction method DM3, in Algorithm 6.

Algorithm 6 sets the upper limit of the number of deleted nodes to *d*_max_ (Algorithm 6, lines 8–9). DM3 first selects nodes to be deleted from each cluster to construct the deletion candidate list by equation (8) (Algorithm 6, lines 3–6) and then puts all these nodes in the list GDCL (Algorithm 6, line 7). If the number of nodes in GDCL exceeds *d*_max_, *d*_max_ nodes are randomly selected for deletion from the solution (Algorithm 6, line 11).

## 4. Computational Experiments

This section describes the computational experiments that we conducted to evaluate the effectiveness and efficiency of the HA-CCP. We first conducted some preliminary experiments to find suitable parameters for HA-CCP. Then, we compare the result of HA-CCP with the state-of-the-art CCP algorithms: IVNS [29], GVNS [30], SGVNS [30], FITS [32], and DNVNS [31]. Section 4.1 introduces benchmark instances and experimental setup, and Section 4.2 describes the comparative experiments on benchmark instances. Section 4.3 illustrates a comparative experiment based on time-to-target (TTT) analysis method.

### 4.1. Benchmark Instances and Experimental Setup

We conducted experiments to evaluate HA-CCP on 90 benchmark instances (available at http://www.optsicom.es/ccp and [30]), which is commonly used to evaluate algorithms for CCP (see, for instance, [29–32]). It contains four sets: RanReal240, RanReal480, RanReal960, and MDG-a instances.  Table 1 lists the experimental datasets. In the second column, *n* represents the number of nodes of the instance and *p* represents the number of clusters.

We use the following indicators to measure the advantages of each algorithm:

#Best/Avg counted the number of instances where a specific algorithm outperforms the other algorithms in terms of the best and the average objective value.

Average Dev_best_/Dev_avg _ indicates the average percent deviation of a specific algorithm's best/average result from the best solution obtained in all algorithms participating in the comparison, where Dev_best_ =((*f*^*∗*^ − *f*_best_)/*f*^*∗*^) ×  100 and Dev_avg_ =((*f*^*∗*^ − *f*_avg_)/*f*^*∗*^) × 100, and *f*_best_  is the best result of each algorithm,  *f*_avg_ is the average result of each algorithm, and *f*^*∗*^ is the best solution obtained by participating in the comparison of all algorithms.

Avg Time recorded the average calculation time (in seconds) required for a particular algorithm to reach its final objective function value.

*p* value_best_/*p* value_avg_ represents the *p* value of the best/average result obtained through the pairwise Wilcoxon statistical test.

Note that, in Section 4.2, we use the result of IVNS, GVNS, SGVNS, FITS, and NDVNS present in [31]. The experiments of IVNS, GVNS, SGVNS, and FITS were executed on an Intel E5-2670 Processor (2.8 GHz) with 2 *G* Byte RAM running under Linux. HA-CCP is implemented in *C*++, compiled with the *g*++ compiler using the option “-O3,” and carried out on a server under Ubuntu Linux (version 16.04) with 1 Core of an Intel Xeon (Cascade Lake) Platinum 8269CY 2.5 GHz CPU and 2 *G* Byte RAM. These results are obtained by running each instance independently 20 times with the running time set to *n* seconds, where *n* is the number of nodes in a given instance.

The HA-CCP has five parameters: the parameter *α* used to balance randomness and greed in CM2, the parameter *δ* that controls the size of the deleted candidate list in DM3, the percentage *β* of deleted nodes in the candidate list of each cluster, and the upper limit of the number of deleted nodes *d*_max_ in DM3 and parameter *ε* (for skewed moves) in HA-CCP. For *α*, *δ*,  and *β*, we conducted the test experiment on 13 instances with different characteristics in RanReal240, RanReal480, RanReal960, and MDG-a. For *α*, we keep the other parameters unchanged and change the value of *α* from 0.2 to 0.8 in steps of 0.2. The best value is obtained when *α* = 0.6. For *δ* and *β*, we use a similar method to obtain *δ*  = 0.7 and *β*  = 0.1. The value settings of parameters (*α*, *δ*, *β*) are consistent with the literature [26], so we did not report the results of this experiment.

For *d*_max_, we test it in the range of {20, 24, 30, 40, 60}. We conducted this experiment on 9 instances with different characteristics in RanReal960 and MDG-a. The statistical results of the experiment are shown in Table 2. In terms of the best objective function value and average objective function value (#Best/Avg), HA-CCP obtains the best value at *d*_max_=24, including obtaining 4 best objective function values and 6 best average values. According to the average percentage deviation (Avg Dev_best_/Dev_avg _), HA-CCP has the smallest deviation of the best solution obtained when *d*_max_=24 (0.02%/0.15%), and there is not much difference between the parameter values in terms of average time. Therefore, we set *d*_max_=24.

For parameter *ε*, according to [30], *ε*=0.01. On this basis, we test *ε*=0.01 and *ε* = 0.005 on 4 instances from RanReal with *p* ≤ 12 and 13 instances from RanReal240, RanReal480, RanReal960, and MDG-a with *p* > 12. The test results are shown in Tables 3 and 4. On the instances with *p* ≤ 2, the effect of *ε*=0.01 is better than *ε* = 0.005. In the other instance, *ε* = 0.005 presents a better effect. Therefore, in instances where the number of clusters is greater than 12, we set  *ε* = 0.005, and in other instances, we set  *ε* = 0.01.

### 4.2. Algorithm Comparison Experiment

To evaluate the performance of HA-CCP, we compared the solution of HA-CCP, IVNS [29], GVNS [30], SGVNS [30], FITS [32], and NDVNS [31]. The instances in the experiment are divided into small and medium instances and large-scale instances.

The experimental statistical results on the datasets of small and medium instances (RanReal240 and RanReal480) are shown in Table 5. Tables 6 and 7 give detailed results. In Table 5, HA-CCP is better than other algorithms on 19 instances in terms of the best objective function value and has better performance on 36 instances from the average result. According to the average percentage deviation, HA-CCP has the smallest deviation from the best solution obtained in the experiment (0.02%/0.13%). The statistical test shows a significant performance difference between HA-CCP and other comparison algorithms (*p* − value  ≤  0.05), except for the best results of NDVNS.

The experimental statistical results on the dataset of large-scale instances (RanReal960) are shown in Table 8. The detailed results are given in Tables 9 and 10. According to Table 8, HA-CCP has obvious advantages in average results. It obtained 20 best average results out of 30 instances and 11 best results. HA-CCP surpasses other comparison algorithms in terms of the average percent deviation of average result from best-found solutions (AvgeDev_avg_=0.19). The *p* value_best_/*p* value_avg_ row shows a statistically significant difference in performance between HA-CCP and all the reference algorithms except NDVNS.

The experimental statistical results on the dataset of large-scale instances (MDG-a) are presented in Table 11. Tables 12 and 13 give detailed results. It can be seen from Table 7 that NDVNS has obvious advantages in solution quality, but HA-CCP is better than other algorithms (such as IVNS, GVNS, SGVNS, and FITS), which is also proved by the results of statistical testing.

From the above experimental results, it can be seen that (1) the average solving efficiency of HA-CCP on all instances is better than all comparison algorithms except NDVNS (because NDVNS does not give the solution time). (2) In terms of solution quality, HA-CCP obtained 58 best average solutions and 30 best solutions on 90 instances, which is better than all comparison algorithms in average results. This shows that HA-CCP has better solution stability. (3) The solution quality of HA-CCP on MDG-a instances is inferior to NDVNS. Therefore, the improvement of HA-CCP combined with NDVNS deserves further study.

### 4.3. Time-to-Target Analysis

**Figure 1:**
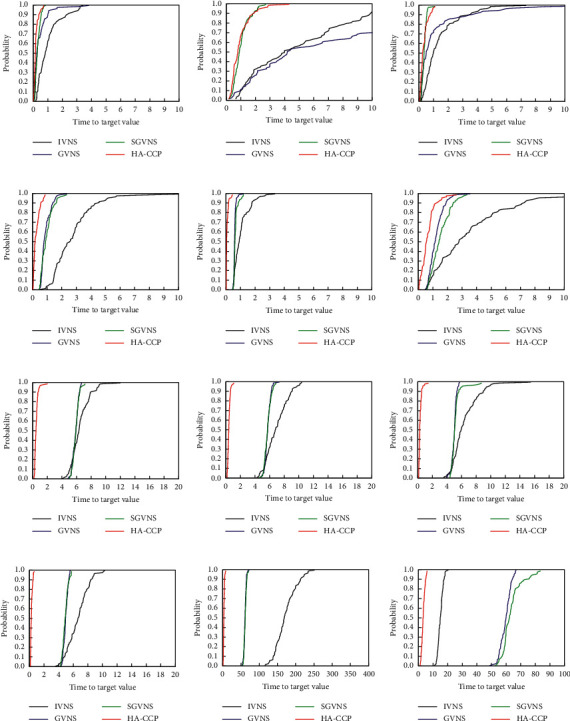
Probability distribution of the time required to reach the value of the objective function. (a) Instance: RanReal240_05 and target:193000. (b) Instance: RanReal240_09 and target: 207700. (c) Instance: RanReal240_16 and target: 202000. (d) Instance: RanReal480_05 and target: 473000. (e) Instance: RanReal480_14 and target: 500. (f) Instance: RanReal480_18 and target: 515000. (g) Instance: RanReal960_02.30 and target: 1380000. (h) Instance: RanReal960_06.30 and target: 1370000. (i) Instance: RanReal960_07.40 and target: 1000000. (j) Instance: RanReal960_05.60 and target: 710000. (k) Instance: MDG-a_23 and target: 360000. (l) Instance: MDG-a_35 and target: 330000.

To further evaluate the efficiency of the HA-CCP algorithm, we apply the time-to-target (TTT) analysis [37], which identifies the empirical probability distribution of the time required to reach a given objective function value. In this experiment, we choose algorithms with known good performance to compare with HA-CCP: VNS, GVNS, and SGVNS. We used the source code of IVNS (http://www.info.univ-angers.fr/pub/hao/ccp.html), GVNS, and SGVNS (http://www.mi.sanu.ac.rs/∼nenad/ccp/), which can be found online. The instance we chose in the experiment is the same as the test instance in [32], and the objective function value recommended in [32] is used. We carried out TTT experiments under the calculation conditions described in Section 4.2 by executing 100 times on each instance for each algorithm. For each instance/target pair, the running time is sorted in ascending order, and the probability associated with the *i*th sorted running time *t*_*i*_ is *p*_*i*_=(*i* − 0.5)/100, and the points (*t*_*i*_, *p*_*i*_) are drawn.  Figure 1 shows the experimental results. The abscissa of the figure represents the time-to-target value in seconds (*s*). The maximum coordinate value of the abscissa is set to a different value to make the comparisons clearer. It can be seen from Figure 1 that in Figures 1(a)–1(f), HA-CCP and SGVNS have similar performance. HA-CCP has the shortest calculation time in the remaining instances.

## 5. Analysis of the Main Components of HA-CCP

In this section, we have studied the contribution of the main components of HA-CCP to performance. We give experimental results from four aspects: “construct solution,” “destruct partial solution,” “destruct and reconstruct partial solution,” and “acceptance criterion for skew” to show that the method used in HA-CCP is better. All experiments in this section were performed under the experimental conditions described in Section 4.1.

### 5.1. Benefit of CM2

In HA-CCP, CM2 is first used to construct the initial feasible solution (Algorithm 2, line 2) and then reconstruct the solution in the iterative process (Algorithm 2, line 9). Different from CM [26] and random construction method (RCM, used in [29, 31, 32]), CM2 can increase the probability of constructing a feasible solution when the upper and lower bounds are tighter and improve the performance of HA-CCP. Therefore, we conducted comparative experiments in the following two aspects.

First, we compared the success rate of constructing the initial feasible solution between RCM and CM2 under different upper and lower bound constraints on 10 RanReal960 instances with *p*=60. In the experiment, the upper and lower bounds *U*_*g*_ and *L*_*g*_ of the instance are adjusted to *U*_*g*_′=*s*_2_ × *U*_*g*_ and *L*_*g*_′=*s*_1_ × *L*_*g*_, respectively. Obviously, *s*_2_ < 1 and *s*_1_ > 1 will tighten the constraints of CCP. For each instance, RCM and CM2 are run 100 times under the new upper and lower bound constraints, respectively, and the success rate of obtaining the initial solution is shown in Table 14. The “average” row indicates the average value of the construction success rate of all the test instances.

We can see from Table 14 that if the upper and lower bounds of the cluster capacity become stricter, the success rate of RCM and CM2 in constructing the initial solution decreases, but the success rate of CM2 is higher than that of RCM. From the average point of view, the success rate of CM2 in the experiment is higher than that of RCM, even as high as 2 times (the last column of Table 14). Therefore, CM2 has better usability than RCM, and it can construct feasible solutions in tighter constrained CCPs.

Secondly, we use RCM to replace CM2 in HA-CCP (denoted as HA-CCP-RCM) for comparison experiments with HA-CCP. The experiment was conducted on the same 10 instances as Section 4.3, and each instance was run 20 times.  Table 15 summarizes the comparison results, and Tables 16 and 17 give detailed results. It can be seen from Table 15 that when the average calculation time is almost the same, HA-CCP with CM2 can obtain more best solution and best average solution. Therefore, CM2 can effectively improve the CCP solution quality.

### 5.2. Benefit of DM3

DM2 [26] is the destruction method used in IG-GRASP [26] to partial destruction solution. To verify the superiority of DM3 used in HA-CCP, we replaced the DM3 in HA-CCP with DM2 and compared the results of HA-CCP using different destruction methods (DM2 and DM3) on the results of the RanReal960 dataset.  Table 18 summarizes the statistical comparison results, and Tables 19 and 20 give detailed results.

As shown in Table 18, DM3 has obtained 29 best solutions and 30 best average objective function value and has a smaller value of Average Dev_best_/Dev_avg _. The Wilcoxon statistical test shows that the best performance and average performance of DM2 and DM3 on RanReal960 are statistically significantly different. Therefore, we can conclude that DM3 is superior to DM2 on RanReal960.

### 5.3. Benefit of the Destruct and Reconstruct Partial Solution

Algorithms IVNS, GVNS, SGVNS, and FITS for CCP used a completely random shaking procedure to shake the current solution, but HA-CCP uses the method of destroying and reconstructing partial solutions. To verify the advantage of the destruct and reconstruct partial solution, we combined HA-CCP with the random shaking procedure used in SGVNS [30], called HA-CCP-RS. We compared the result of HA-CCP with HA-CCP-RS on the MDG-a dataset.  Table 21 describes the statistical comparison results, and Tables 22 and 23 give detailed results. According to Table 21, HA-CCP has a better performance than HA-CCP-RS in all indicators.

### 5.4. Benefit of Acceptance Criterion for Skew

HA-CCP added an acceptance criterion for skewing the search to other promising areas of the solution space. To verify the performance of acceptance criterion for skew to the performance, we compared the result of HA-CCP with the HA-CCP without acceptance criterion for skew (HA-CCP-NAC for short) on RanReal240 and RanReal480 datasets.  Table 24 shows the statistical comparison results, and detailed results are given in Tables 25 and 26. As shown in Table 24, HA-CCP has a better performance than HA-CCP-NAC in best results and average results.

In summary, from the experimental results of the four aspects of “the construction of solution,” “the destruction of the partial solution,” “the destruction and reconstruction of the partial solution,” “the acceptance criteria,” no matter which component is replaced, the overall performance of the algorithm decreases significantly. Therefore, we believe that the combination of these components has obtained good experimental results. The effective combination of these components helps the algorithm jump out of the local optimum and obtains a better balance between intensification and diversification levels.

## 6. Conclusion

The capacitated clustering problem (CCP) has a wide range of applications. In this study, we propose a hybrid heuristic algorithm HA-CCP for CCP. After constructing the initial solution, HA-CCP partially destructs and reconstructs the current solution through a combination of greediness and randomness to obtain a new solution. On this basis, an acceptance criterion is added, which allows the current solution to be skewed to move to inferior solutions so that more promising solutions can be explored in the solution space.

The competitive experiments on the benchmark instances show that HA-CCP is superior to all comparison algorithms except NVSD in terms of average solving efficiency for all instances (because the solution time is not given). The result of the time-to-target analysis also verifies the efficiency of HA-CCP. Moreover, HA-CCP is better than all comparison algorithms because the best average solution of 58 instances is obtained on all 90 instances, which shows that HA-CCP has better solution stability. However, the solution quality of HA-CCP on MDG-a instances is obviously inferior to NDVNS.

As future work, we believe that the following research is worthwhile. First, the automatic parameter adjustment tool irace is used [38] to adjust the HA-CCP parameters to find a better parameter configuration. Second, the improved solution construction strategy enables the algorithm to solve the more restrictive CCP. Third, NDVNS is combined to improve HA-CCP, so that it has better solution quality on CCP including MDG-a.

## Figures and Tables

**Algorithm 1:**
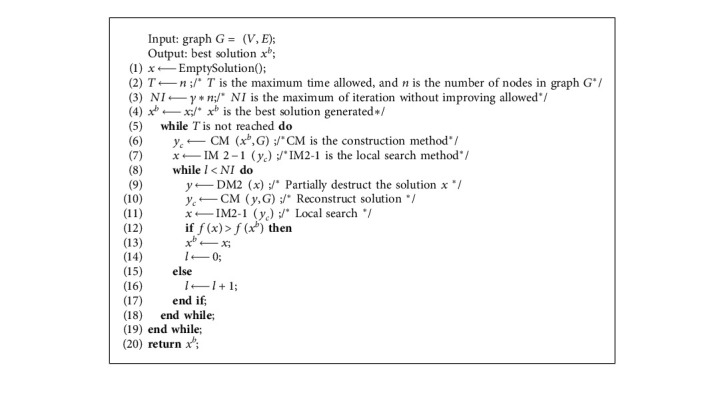
IG-GRASP.

**Algorithm 2:**
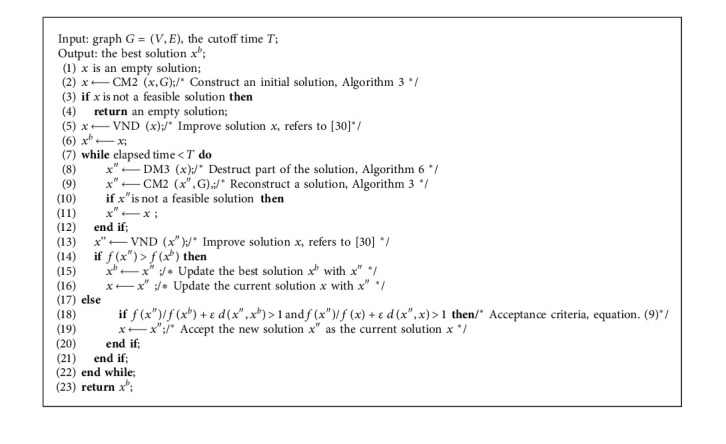
HA-CCP.

**Algorithm 3:**
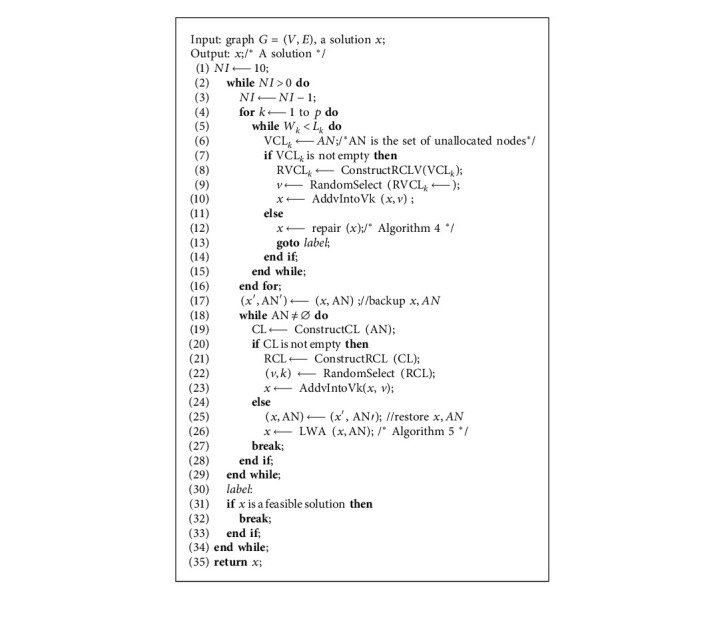
CM2 /^*∗*^Construction method^*∗*^/.

**Algorithm 4:**
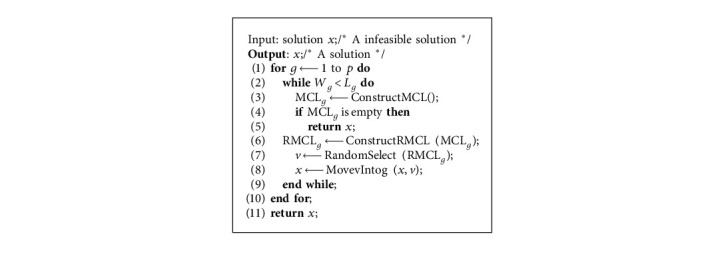
Repair.

**Algorithm 5:**
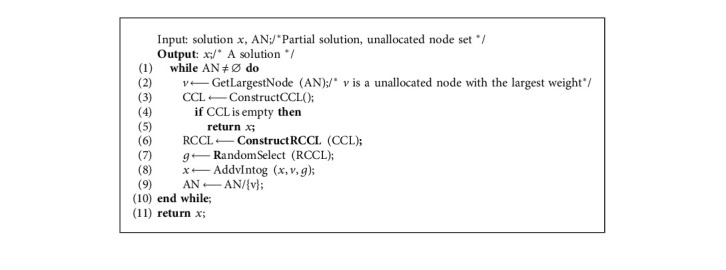
LWA.

**Algorithm 6:**
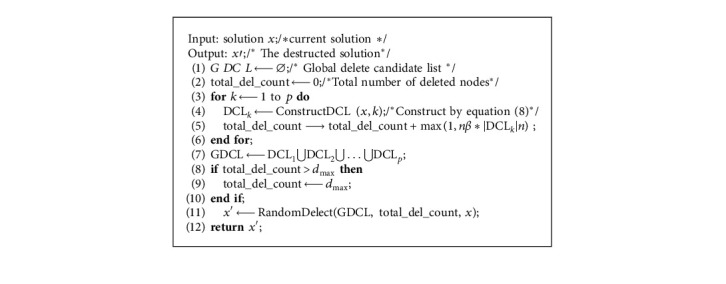
DM3 /∗Destruction method∗/.

**Table 1:** Experimental dataset.

Dataset name	Feature	Number of instances
RanReal240	*n*=240 and *p*=12	20
RanReal480	*n*=480 and *p*=20	20
RanReal960	*n*=960 and 30 ≤ *p* ≤ 60	30
MDG-a	*n*=2000 and *p*=50	20

**Table 2:** Best *d*_max_ parameter values identified for HA-CCP.

*d*_max_	20	24	30	40	60
#Best/Avg	3/3	**4/6**	0/0	0/0	0/0
*p* value_best_/*p* value_avg_	1.00/0.004		0.18/0.004	0.004/0.004	0.004/0.004
Avg Dev_best_/Dev_avg_ (%)	0.04/0.18	**0.02/0.15**	0.04/0.19	0.11/0.24	0.17/0.29
AvgTime (s)	1074.01	1062.57	1051.71	1037.85	**1012.09**

**Table 3:** Best *ε* parameter values identified for HA-CCP on the instances with *p* ≤ 12.

*ε*	0.01	0.005
#Best/Avg	**4/4**	1/0
*p* value_best_/*p* value_avg_	0.25/0.125	
Avg Dev_best_/Dev_avg_ (%)	**0.00/0.05**	0.03/0.18
AvgTime (s)	118.23	**108.86**

**Table 4:** Best *ε* parameter values identified for HA-CCP on the instances with *p* > 12.

*ε*	0.01	0.005
#Best/Avg	2/0	**11/13**
*p* value_best_/*p* value_avg_	0.022/2.4*E* − -4	
Avg Dev_best_/Dev_avg_ (%)	0.10/0.24	**0.007/0.16**
AvgTime (s)	**807.47**	837.90

**Table 5:** Experimental statistical results on RanReal240 and RanReal480.

	IVNS	GVNS	SGVNS	FITS	NDVNS	HA-CPP
#Best/Avg	3/0	0/0	2/1	8/0	**19**/3	**19**/**36**
*p* value_bes*t*_/*p* value_avg_	3.46*E* − 07/2.54*E* − 10	2.54*E* − 10/2.54*E* − 10	2.47*E* − 05/1.25*E* − 8	2.46*E* − 04/1.25*E* − 08	0.87/7.62*E* − 08	
Avg Dev_best_/Dev_avg_ (%)	0.17/0.35	0.35/0.63	0.08/0.19	0.09/0.27	0.03/0.25	**0.02**/**0.13**
AvgTime (s)	280.01	296.66	226.89	249.39	—	**217.25**

*Note*. In Table 5, the symbol “—” denotes the cases when the result is not reported in the literature.

**Table 6:** Comparative results of different algorithms on RanReal240 and RanReal480.

Instance name	IVNS	GVNS	SGVNS	FITS	NDVNS	HA-CCP
RanReal240_01	224831.56/224571.29	224580.56/224207.09	224968.01/224769.72	224941.48/224802.06	224949.51/224689.70	**225003.70/224897.01**
RanReal240_02	**204624.36**/204275.49	204205.41/203682.12	**204624.36**/204444.31	**204624.36**/204359.38	204563.81/204214.81	**204624.36/204515.06**
RanReal240_03	198861.68/198606.95	198472.78/197806.87	199059.56/198849.22	198954.91/198799.84	198976.88/198681.37	**199079.37/198915.84**
RanReal240_04	225390.88/225069.14	225144.59/224463.81	225627.16/**225389.88**	225627.16/225364.97	225618.17/225185.91	**225683.17**/225346.54
RanReal240_05	195540.41/195184.84	194911.72/194361.91	195516.57/195306.51	**195564.48**/195320.28	195539.89/195401.63	195540.41/**195469.00**
RanReal240_06	216713.91/216355.53	216383.13/215849.06	216733.31/216584.23	**216747.32**/216487.02	216747.32/216173.65	216730.26/**216613.93**
RanReal240_07	209216.90/208992.44	209118.64/208329.21	209223.34/209080.57	**209305.70**/209029.23	209273.70/209063.40	209282.88/**209150.41**
RanReal240_08	**205246.82**/204842.79	204754.36/204137.78	205154.20/204951.2	**205246.82**/204961.05	**205246.82**/205099.31	**205246.82/205110.51**
RanReal240_09	209142.07/208720.16	208702.16/208276.52	209007.44/208904.78	209159.16/208952.48	**209186.90/208987.58**	209057.64/208897.23
RanReal240_10	192885.48/192598.79	192343.75/191874.34	**193062.60**/192842.05	192986.21/192811.13	192948.92/192523.02	193044.16/**192954.72**
RanReal240_11	204647.20/204377.08	204399.04/203900.12	204615.71/204480.92	**204722.75**/204559.39	**204722.75**/204398.23	**204722.75/204638.07**
RanReal240_12	201028.32/200763.75	200822.69/200150.52	201076.30/200938.30	**201117.11**/200797.67	200978.99/200737.16	**201117.11/201006.45**
RanReal240_13	202331.20/202027.28	201977.87/201356.20	202321.58/202198.63	202335.99/202139.57	202345.12/202276.80	**202345.48/202285.83**
RanReal240_14	228870.89/228520.04	228661.60/228054.32	228775.14/228569.78	228870.89/228554.78	228870.89/228396.82	**228971.03/228740.72**
RanReal240_15	191152.17/190827.68	190575.48/189965.59	191238.53/191058.62	191255.87/190923.28	**191263.28**/190945.28	191243.76/**191117.60**
RanReal240_16	**204074.95**/203668.49	203816.48/203270.11	203991.53/203649.04	204054.99/203710.39	**204074.95**/203918.76	204072.57/**203961.38**
RanReal240_17	195206.73/194950.57	194840.79/194404.70	195423.83/195241.19	**195561.36**/195243.32	195509.13/195236.75	195393.97/**195278.93**
RanReal240_18	194916.37/194704.23	194915.62/194114.09	195120.98/194967.73	195100.39/194872.13	**195167.14**/194886.16	**195167.14/195069.80**
RanReal240_19	199200.03/198905.05	198828.82/198119.14	199307.33/199093.86	199225.98/199040.43	199216.46/198895.83	**199307.33/199204.90**
RanReal240_20	212264.10/211871.74	211984.80/211458.51	212268.46/212037.43	212268.52/212049.85	**212323.22/212130.13**	212229.46/212111.19
RanReal480_01	554337.23/553795.85	553224.53/552326.59	555430.60/554994.68	555489.92/554376.54	**556639.68/555566.55**	556126.86/555338.06
RanReal480_02	510066.41/509058.46	508711.62/507540.62	510718.79/510304.78	511280.50/509757.15	**511666.95**/510495.78	511566.55/**510924.27**
RanReal480_03	496334.51/495409.98	495140.23/493706.92	497725.86/496785.80	497295.19/496059.50	497846.57/495871.46	**498028.54/497109.59**
RanReal480_04	521669.00/520051.55	520653.34/519051.60	522572.81/521952.28	522305.16/521062.13	522748.49/520295.34	**522790.22/521999.31**
RanReal480_05	483670.19/482390.51	481803.95/480508.80	483819.77/482603.84	484084.66/482867.74	484742.51/482831.18	**485138.44/484092.59**
RanReal480_06	533589.61/532462.25	532702.72/531627.12	534515.67/533916.00	533991.27/533036.36	**535503.61**/533138.78	534961.79/**534114.47**
RanReal480_07	545343.81/544060.83	544445.60/542318.56	545812.49/545302.82	545470.73/544651.12	**546951.97**/545548.47	546503.90/**545582.87**
RanReal480_08	531974.48/531023.25	531287.92/529525.39	532736.12/532109.62	532417.42/531667.91	**533098.56**/531631.32	532891.23/**532161.08**
RanReal480_09	555604.38/554820.43	555163.27/553394.39	556865.18/556081.91	556868.85/555634.40	**557283.90**/555998.60	557120.25/**556265.26**
RanReal480_10	519066.57/518412.32	517431.34/516228.20	520014.70/518024.16	520257.54/518071.71	520481.15/518812.29	**520492.06/519891.18**
RanReal480_11	523463.33/522201.99	522626.50/521218.97	524124.60/523508.02	523991.29/522816.94	524059.91/522416.53	**524669.99/523760.48**
RanReal480_12	501462.57/500055.30	499914.17/498596.97	502570.10/501632.20	501915.56/500776.79	502656.68/501171.81	**503359.96/502069.60**
RanReal480_13	534294.24/533478.80	533672.27/532322.68	535411.94/534651.24	535025.51/533823.79	**535633.80**/533605.55	535251.73/**534707.51**
RanReal480_14	513186.65/512501.71	512764.33/511212.78	514537.52/513935.11	514107.62/513053.25	514696.90/512600.42	**515476.42/514801.49**
RanReal480_15	516657.20/515416.17	515607.47/514465.20	518029.18/517189.00	517205.02/516018.38	**518605.32**/516455.65	518370.90/**517283.29**
RanReal480_16	549230.25/548274.66	549033.57/547075.83	549840.64/549253.54	549552.63/548462.13	**550482.48**/549108.44	550317.71/**549881.85**
RanReal480_17	537223.44/536149.06	536402.45/534770.20	537993.79/537568.15	537924.55/536745.39	538331.26/536829.04	**538745.70/537826.26**
RanReal480_18	525490.09/524515.11	524631.54/522908.72	526349.49/525453.50	525822.76/524712.42	**526466.23**/523932.37	526314.82/**525702.47**
RanReal480_19	522280.40/521442.89	521672.59/519720.44	522757.15/522218.13	522316.22/521267.22	**523219.84**/522114.62	522958.23/**522365.10**
RanReal480_20	518436.63/516935.15	516488.64/515450.41	518847.01/518202.52	518349.10/517430.77	**519492.21**/518475.86	519277.92/**518518.27**
#Best	3/0	0/0	2/1	8/0	**19**/3	**19/36**
*p* Value	3.46*E* − 07/2.54*E* – 10	2.54*E* − 10/2.54*E* − 10	2.47*E* − 05/1.25*E* − 08	2.46*E* − 04/1.25*E* − 08	0.87/7.62*E* − 08	

*Note*. In Table 6, data A/B denote the best objective value and the average objective value of the instance found by the algorithm.

**Table 7:** Dev_best_, Dev_avg_, and average time of different algorithms on RanReal240 and RanReal480.

Instance name	IVNS	GVNS	SGVNS	FITS	DNVNS	HA-CCP
RanReal240_01	0.08/0.19/155.57	0.19/0.35/155.92	0.02/0.10/127.16	0.03/0.09/149.09	0.02/0.14/-	0.00/0.05/127.88
RanReal240_02	0.00/0.17/184.44	0.2/0.46/159.56	0.00/0.09/158.20	0.00/0.13/150.75	0.03/0.20/-	0.00/0.05/111.58
RanReal240_03	0.11/0.24/186.06	0.3/0.64/164.11	0.01/0.12/139.26	0.06/0.14/169.95	0.05/0.20/-	0.00/0.08/135.66
RanReal240_04	0.13/0.27/159.70	0.24/0.54/175.00	0.02/0.13/137.87	0.02/0.14/118.58	0.03/0.22/-	0.00/0.15/124.67
RanReal240_05	0.01/0.19/155.02	0.33/0.61/179.73	0.02/0.13/136.48	0.00/0.12/132.33	0.01/0.08/-	0.01/0.05/135.27
RanReal240_06	0.02/0.18/174.51	0.17/0.41/187.59	0.01/0.08/126.61	0.00/0.12/155.99	0.00/0.26/-	0.01/0.06/129.28
RanReal240_07	0.04/0.15/160.67	0.09/0.47/195.33	0.04/0.11/144.35	0.00/0.13/144.96	0.02/0.12/-	0.01/0.07/127.25
RanReal240_08	0.00/0.20/145.94	0.24/0.54/193.88	0.05/0.14/122.79	0.00/0.14/124.22	0.00/0.07/-	0.00/0.07/112.98
RanReal240_09	0.02/0.22/194.36	0.23/0.44/171.09	0.09/0.13/136.24	0.01/0.11/141.69	0.00/0.10/-	0.06/0.14/98.89
RanReal240_10	0.09/0.24/183.49	0.37/0.62/145.39	0.00/0.11/162.77	0.04/0.13/134.20	0.06/0.28/-	0.01/0.06/111.58
RanReal240_11	0.04/0.17/157.98	0.16/0.40/155.12	0.05/0.12/136.25	0.00/0.08/129.91	0.00/0.16/-	0.00/0.04/125.15
RanReal240_12	0.04/0.18/186.71	0.15/0.48/157.43	0.02/0.09/123.13	0.00/0.16/132.03	0.07/0.19/-	0.00/0.06/108.32
RanReal240_13	0.01/0.16/173.46	0.18/0.49/171.44	0.01/0.07/127.25	0.00/0.10/131.34	0.00/0.03/-	0.00/0.03/139.97
RanReal240_14	0.04/0.20/147.56	0.14/0.40/164.87	0.09/0.18/112.27	0.04/0.18/135.87	0.04/0.25/-	0.00/0.10/122.04
RanReal240_15	0.06/0.23/167.88	0.36/0.68/188.75	0.01/0.11/154.19	0.00/0.18/131.83	0.00/0.17/-	0.01/0.08/101.57
RanReal240_16	0.00/0.20/165.22	0.13/0.39/181.82	0.04/0.21/130.93	0.01/0.18/89.11	0.00/0.08/-	0.00/0.06/102.24
RanReal240_17	0.18/0.31/171.75	0.37/0.59/179.25	0.07/0.16/149.06	0.00/0.16/171.03	0.03/0.17/-	0.09/0.14/132.10
RanReal240_18	0.13/0.24/154.60	0.13/0.54/169.52	0.02/0.10/167.15	0.03/0.15/152.83	0.00/0.14/-	0.00/0.05/110.87
RanReal240_19	0.05/0.20/173.65	0.24/0.60/179.99	0.00/0.11/140.21	0.04/0.13/98.09	0.05/0.21/-	0.00/0.05/121.71
RanReal240_20	0.03/0.21/188.64	0.16/0.41/175.52	0.03/0.13/112.33	0.03/0.13/115.38	0.00/0.09/-	0.04/0.10/99.55
RanReal480_01	0.41/0.51/391.12	0.61/0.77/419.98	0.22/0.30/301.25	0.21/0.41/340.60	0.00/0.19/-	0.09/0.23/349.71
RanReal480_02	0.31/0.51/403.38	0.58/0.81/414.24	0.19/0.27/293.36	0.08/0.37/355.59	0.00/0.23/-	0.02/0.15/330.24
RanReal480_03	0.34/0.53/420.24	0.58/0.87/427.12	0.06/0.25/331.81	0.15/0.40/352.06	0.04/0.43/-	0.00/0.18/332.28
RanReal480_04	0.21/0.52/383.19	0.41/0.72/428.09	0.04/0.16/332.13	0.09/0.33/399.51	0.01/0.48/-	0.00/0.15/329.59
RanReal480_05	0.30/0.57/417.55	0.69/0.95/448.15	0.27/0.52/325.05	0.22/0.47/335.84	0.08/0.48/-	0.00/0.22/301.91
RanReal480_06	0.36/0.57/393.37	0.52/0.72/428.17	0.18/0.30/300.77	0.28/0.46/390.06	0.00/0.44/-	0.10/0.26/280.00
RanReal480_07	0.29/0.53/395.01	0.46/0.85/423.31	0.21/0.30/342.07	0.27/0.42/390.23	0.00/0.26/-	0.08/0.25/285.02
RanReal480_08	0.21/0.39/388.45	0.34/0.67/398.32	0.07/0.19/356.39	0.13/0.27/380.64	0.00/0.28/-	0.04/0.18/283.23
RanReal480_09	0.30/0.44/376.21	0.38/0.70/421.61	0.08/0.22/350.35	0.07/0.30/383.67	0.00/0.23/-	0.03/0.18/255.35
RanReal480_10	0.27/0.40/383.71	0.59/0.82/428.06	0.09/0.47/300.43	0.05/0.47/328.85	0.00/0.32/-	0.00/0.12/307.38
RanReal480_11	0.23/0.47/383.91	0.39/0.66/421.15	0.10/0.22/320.08	0.13/0.35/383.65	0.12/0.43/-	0.00/0.17/299.74
RanReal480_12	0.38/0.66/397.43	0.68/0.95/409.11	0.16/0.34/270.86	0.29/0.51/347.32	0.14/0.43/-	0.00/0.26/349.86
RanReal480_13	0.25/0.40/404.23	0.37/0.62/390.76	0.04/0.18/327.70	0.11/0.34/330.84	0.00/0.38/-	0.07/0.17/333.59
RanReal480_14	0.44/0.58/405.04	0.53/0.83/419.87	0.18/0.30/319.14	0.27/0.47/352.07	0.15/0.56/-	0.00/0.13/340.90
RanReal480_15	0.38/0.61/384.96	0.58/0.80/418.77	0.11/0.27/313.91	0.27/0.50/387.02	0.00/0.41/-	0.05/0.25/282.23
RanReal480_16	0.23/0.40/345.11	0.26/0.62/419.82	0.12/0.22/301.16	0.17/0.37/392.78	0.00/0.25/-	0.03/0.11/294.16
RanReal480_17	0.28/0.48/386.16	0.43/0.74/411.52	0.14/0.22/320.59	0.15/0.37/360.93	0.08/0.36/-	0.00/0.17/336.43
RanReal480_18	0.19/0.37/368.20	0.35/0.68/431.30	0.02/0.19/278.26	0.12/0.33/320.60	0.00/0.48/-	0.03/0.15/330.79
RanReal480_19	0.18/0.34/400.78	0.30/0.67/435.58	0.09/0.19/306.80	0.17/0.37/380.69	0.00/0.21/-	0.05/0.16/343.32
RanReal480_20	0.20/0.49/385.30	0.58/0.78/420.08	0.12/0.25/339.09	0.22/0.40/353.52	0.00/0.20/-	0.04/0.19/345.87
Average	0.17/0.35/280.01	0.35/0.63/296.66	0.08/0.19/226.89	0.09/0.27/249.39	0.03/0.25/-	0.02/0.13/217.25

*Note*. In Table 7, data A/B/C denote the value of Dev_best_, the value of Dev_avg_, and the algorithm's average time to find a final solution. The symbol “-” denotes the cases when the result is not reported in the literature.

**Table 8:** Experimental statistical results on RanReal960.

	IVNS	GVNS	SGVNS	FITS	NDVNS	HA-CPP
#Best/Avg	0/0	0/0	0/0	1/2	**18**/10	11/**18**
*p* value_best_/*p* value_avg_	4.32*E* − 08/4.32*E* − 08	4.32*E* − 08/4.32*E* − 08	4.32*E* − 08/4.32*E* − 08	1.18*E* − 05/1.18*E* − 05	0.14/0.07	
Avg Dev_best_/Dev_avg_ (%)	0.81/1.02	0.81/1.12	0.19/0.39	0.52/0.62	**0.04**/0.24	**0.04**/**0.19**
AvgTime (s)	843.9	862.2	840.91	893.11	—	**723.82**

*Note*. In Table 8, the symbol “—” denotes the cases when the result is not reported in the literature.

**Table 9:** Comparative results of different algorithms on RanReal960.

Instance name	IVNS	GVNS	SGVNS	FITS	NDVNS	HA-CCP
RanReal960_01.30	1331323.28/1329095.10	1331996.73/1328415.40	1337853.23/1335679.54	1333878.00/1332712.78	**1340369.47/1338452.93**	1339904.73/1337999.56
RanReal960_02.30	1426870.24/1423903.72	1427037.60/1422960.00	1433071.63/1430808.90	1434529.49/**1433886.30**	**1435819.84**/1433258.34	1435087.46/1433503.63
RanReal960_03.30	1390084.19/1387508.88	1390367.98/1386753.98	1395846.80/1394012.26	1392101.18/1390924.21	**1398554.78/1397154.11**	1397520.91/1396135.59
RanReal960_04.30	1407607.65/1405972.04	1406916.02/1403198.73	1413478.82/1410438.07	1414344.67/1412460.01	**1414919.86/1412464.18**	1413878.60/1411928.57
RanReal960_05.30	1363405.33/1360883.59	1362240.30/1359314.24	1370560.76/1367959.84	1365975.96/1365612.74	**1372686.88/1370958.72**	1371391.13/1369452.03
RanReal960_06.30	1413074.62/1409803.6	1410969.99/1408670.37	1417338.38/1415708.82	1413476.58/1412750.08	1420632.38/**1419467.40**	**1420942.81**/1418222.26
RanReal960_07.30	1332205.25/1329503.04	1332748.04/1329170.21	1339735.87/1337029.01	1334504.35/1334263.93	**1341829.68/1340275.06**	1341640.99/1340054.65
RanReal960_08.30	1462280.35/1458524.76	1460407.11/1457531.52	1466738.95/1464441.53	1463737.39/1462602.72	**1469545.99/1466830.82**	1467330.75/1465505.59
RanReal960_09.30	1378445.37/1376305.91	1380206.02/1374713.94	1385287.77/1382960.30	1381577.32/1379280.98	**1387514.11/1385397.31**	1385795.46/1384258.20
RanReal960_10.30	1377646.33/1374311.23	1377009.66/1373790.24	1384154.95/1381870.76	1379905.83/1378772.67	**1386801.94/1384724.79**	1384935.52/1383811.91
RanReal960_01.40	1034548.05/1031462.28	1032872.42/1030512.99	1041148.42/1038487.54	1035642.67/1034626.82	**1042735.72**/1040717.99	1042362.60/**1041082.27**
RanReal960_02.40	1108588.66/1106671.47	1109086.02/1106469.64	1115789.54/1113937.77	1110547.59/1110074.36	1117471.02/1115602.03	**1117552.44/1115723.68**
RanReal960_03.40	1081509.08/1079553.82	1080281.13/1077244.61	1086488.77/1085060.19	1083240.15/1082948.77	**1089012.05**/1087325.53	1088964.98/**1087568.20**
RanReal960_04.40	1096347.39/1092785.74	1096438.26/1091633.05	1100866.71/1098759.10	**1103897.12/1101073.37**	1102222.98/1100256.11	1101734.58/1100608.60
RanReal960_05.40	1056103.80/1054445.69	1057478.73/1054478.20	1063682.56/1062213.71	1059158.09/1058478.46	**1066415.88**/1064390.34	1066157.77/**1065037.50**
RanReal960_06.40	1096895.36/1095721.54	1099861.44/1093932.85	1104590.22/1102651.28	1100368.74/1100064.66	**1107955.38**/1105069.06	1107485.89/**1105353.87**
RanReal960_07.40	1034299.55/1032667.93	1035241.97/1031362.33	1041064.58/1038879.34	1043376.95/1039933.23	**1043921.14**/1041464.08	1043176.75/**1042209.24**
RanReal960_08.40	1137464.75/1136002.24	1136567.63/1133556.70	1142282.86/1141171.70	1139865.05/1138054.46	**1144615.34**/1141877.82	1143818.84/**1142790.55**
RanReal960_09.40	1068288.24/1066496.30	1068892.92/1066427.06	1076229.18/1073798.91	1072116.14/1071426.07	**1076786.59/1075606.23**	1076536.88/1074554.12
RanReal960_10.40	1069556.59/1067889.46	1069985.94/1067548.97	1077400.98/1074400.36	1072919.65/1071480.21	**1079212.31**/1077123.00	1078818.14/**1077728.42**
RanReal960_01.60	726465.37/724244.42	725912.84/723156.84	732096.63/730601.10	727690.58/727222.73	733900.43/731787.78	**733960.17/732785.75**
RanReal960_02.60	770060.9/768413.74	770477.46/768062.71	776289.95/775060.74	773921.97/772572.49	776085.50/774619.17	**777733.23/776084.26**
RanReal960_03.60	753090.65/751419.9	753094.36/750898.37	760248.25/758432.10	756677.95/755442.64	760728.09/758924.49	**761312.30/760046.12**
RanReal960_04.60	762952.42/761387.83	763837.98/760835.94	769112.25/767780.08	765253.27/764696.30	**770170.61**/768057.59	769559.96/**768476.28**
RanReal960_05.60	741248.79/739443.77	741932.85/738830.28	748581.43/746014.20	743715.56/743165.17	748244.40/747006.91	**749316.68/747647.96**
RanReal960_06.60	761947.94/760369.56	762260.95/759048.94	767679.61/765628.88	763029.06/761957.51	768180.66/765223.34	**769233.87/767971.96**
RanReal960_07.60	723439.86/721167.69	723786.02/720360.15	728827.33/727427.19	725993.23/725733.17	731064.84/728984.94	**732249.82/730484.07**
RanReal960_08.60	789552.19/786344.58	787775.07/785553.15	794363.93/792538.90	791334.42/790285.98	794354.56/791262.07	**795313.86/793390.82**
RanReal960_09.60	747509.96/745313.38	746710.78/744341.99	753943.93/751871.94	750858.18/749209.83	754578.20/753033.20	**754811.24/753473.44**
RanReal960_10.60	748812.21/746701.78	747565.74/745004.51	754666.01/752583.88	749883.45/748985.05	755076.90/753154.35	**756020.38/754733.58**
#Best	0/0	0/0	0/0	1/2	**18**/10	11/**18**
*p* value	4.32*E* − 08/4.32*E* − 08	4.32*E* − 08/4.32*E* − 08	4.32*E* − 08/4.32*E* − 08	1.18*E* − 05/1.18*E* − 05	0.14/0.07	

*Note*. In Table 9, data A/B denote the best objective value and the average objective value of the instance found by the algorithm.

**Table 10:** Dev_best_, Dev_avg_, and average time of different algorithms on RanReal960.

Instance name	IVNS	GVNS	SGVNS	FITS	NDVNS	HA-CCP
RanReal960_01.30	0.67/0.84/918.26	0.62/0.89/811.54	0.19/0.35/856.21	0.48/0.57/915.53	0/0.14/-	0.03/0.18/720.64
RanReal960_02.30	0.62/0.83/797.61	0.61/0.90/842.95	0.19/0.35/835.98	0.09/0.13/904.23	0/0.18/-	0.05/0.16/731.68
RanReal960_03.30	0.61/0.79/883.41	0.59/0.84/860.63	0.19/0.32/829.85	0.46/0.55/836.81	0/0.1/-	0.07/0.17/673.02
RanReal960_04.30	0.52/0.63/919.96	0.57/0.83/832.99	0.10/0.32/822.40	0.04/0.17/929.49	0/0.17/-	0.07/0.21/780.14
RanReal960_05.30	0.68/0.86/804.13	0.76/0.97/883.85	0.15/0.34/840.42	0.49/0.52/957.25	0/0.13/-	0.09/0.24/686.23
RanReal960_06.30	0.55/0.78/857.85	0.70/0.86/801.30	0.25/0.37/834.76	0.53/0.58/935.81	0.02/0.1/-	0.00/0.19/777.19
RanReal960_07.30	0.72/0.92/875.58	0.68/0.94/850.22	0.16/0.36/816.60	0.55/0.56/894.98	0/0.12/-	0.01/0.13/769.18
RanReal960_08.30	0.49/0.75/804.15	0.62/0.82/822.22	0.19/0.35/831.19	0.40/0.47/948.47	0/0.18/-	0.15/0.27/686.39
RanReal960_09.30	0.65/0.81/815.06	0.53/0.92/863.40	0.16/0.33/812.81	0.43/0.59/884.89	0/0.15/-	0.12/0.23/641.61
RanReal960_10.30	0.66/0.9/878.66	0.71/0.94/850.02	0.19/0.36/792.14	0.50/0.58/897.68	0/0.15/-	0.13/0.22/775.20
RanReal960_01.40	0.79/1.08/877.73	0.95/1.17/847.87	0.15/0.41/832.62	0.68/0.78/913.51	0/0.19/-	0.04/0.16/724.70
RanReal960_02.40	0.8/0.97/824.06	0.76/0.99/876.23	0.16/0.32/830.26	0.63/0.67/938.57	0.01/0.17/-	0.00/0.16/689.19
RanReal960_03.40	0.69/0.87/905.64	0.80/1.08/850.33	0.23/0.36/816.43	0.53/0.56/829.18	0/0.15/-	0.00/0.13/726.02
RanReal960_04.40	0.68/1.01/800.47	0.68/1.11/904.06	0.27/0.47/761.94	0.00/0.26/931.93	0.15/0.33/-	0.20/0.30/737.53
RanReal960_05.40	0.97/1.12/826.35	0.84/1.12/857.74	0.26/0.39/803.47	0.68/0.74/882.95	0/0.19/-	0.02/0.13/737.69
RanReal960_06.40	1.00/1.10/876.40	0.73/1.27/835.26	0.30/0.48/840.43	0.68/0.71/795.61	0/0.26/-	0.04/0.23/764.48
RanReal960_07.40	0.92/1.08/882.22	0.83/1.20/884.53	0.27/0.48/871.15	0.05/0.38/891.65	0/0.24/-	0.07/0.16/796.50
RanReal960_08.40	0.62/0.75/807.24	0.70/0.97/851.96	0.20/0.30/827.89	0.42/0.57/884.17	0/0.24/-	0.07/0.16/711.17
RanReal960_09.40	0.79/0.96/818.16	0.73/0.96/878.54	0.05/0.28/847.56	0.43/0.50/873.44	0/0.11/-	0.02/0.21/716.20
RanReal960_10.40	0.89/1.05/872.90	0.85/1.08/863.94	0.17/0.45/860.78	0.58/0.72/943.53	0/0.19/-	0.04/0.14/689.14
RanReal960_01.60	1.02/1.32/789.02	1.10/1.47/901.12	0.25/0.46/907.89	0.85/0.92/901.04	0.01/0.3/-	0.00/0.16/751.24
RanReal960_02.60	0.99/1.20/826.25	0.93/1.24/874.07	0.19/0.34/873.06	0.49/0.66/887.61	0.21/0.4/-	0.00/0.21/687.37
RanReal960_03.60	1.08/1.30/808.81	1.08/1.37/852.82	0.14/0.38/873.26	0.61/0.77/923.81	0.08/0.31/-	0.00/0.17/658.15
RanReal960_04.60	0.94/1.14/853.85	0.82/1.21/830.99	0.14/0.31/839.14	0.64/0.71/819.9	0/0.27/-	0.08/0.22/723.40
RanReal960_05.60	1.08/1.32/831.31	0.99/1.40/895.34	0.10/0.44/868.22	0.75/0.82/889.58	0.14/0.31/-	0.00/0.22/691.55
RanReal960_06.60	0.95/1.15/766.03	0.91/1.32/872.94	0.20/0.47/836.76	0.81/0.95/864.12	0.14/0.52/-	0.00/0.16/727.95
RanReal960_07.60	1.20/1.51/918.06	1.16/1.62/909.03	0.47/0.66/878.59	0.85/0.89/875.95	0.16/0.45/-	0.00/0.24/680.08
RanReal960_08.60	0.72/1.13/851.60	0.95/1.23/911.42	0.12/0.35/859.76	0.50/0.63/870.01	0.12/0.51/-	0.00/0.24/761.94
RanReal960_09.60	0.97/1.26/770.73	1.07/1.39/849.73	0.11/0.39/868.30	0.52/0.74/900.64	0.03/0.24/-	0.00/0.18/740.51
RanReal960_10.60	0.95/1.23/855.35	1.12/1.46/898.95	0.18/0.45/857.38	0.81/0.93/870.89	0.12/0.38/-	0.00/0.17/758.65
Average	0.81/1.02/843.9	0.81/1.12/862.2	0.19/0.39/840.91	0.52/0.62/893.11	0.04/0.24/-	0.04/0.19/723.82

*Note*. In Table 10, data A/B/C denote the value of Dev_best_, the value of Dev_avg_, and the algorithm's average time to find a final solution. The symbol “-” denotes the cases when the result is not reported in the literature.

**Table 11:** Experimental statistical results on the MDG-a.

	IVNS	GVNS	SGVNS	FITS	NDVNS	HA-CPP
#Best/Avg	0/0	0/0	0/0	0/0	**20**/**16**	0/4
*p* value_best_/*p* value_avg_	7.74*E* − 06/7.74*E* − 06	7.74*E* − 06/7.74*E* − 06	7.74*E* − 06/7.74*E* − 06	7.74*E* − 06/7.74*E* − 06	7.74*E* − 06/0.01	
Avg *De*v_best_/Dev_avg_ (%)	2.63/3.4	0.99/1.01	0.43/0.57	2.12/2.43	**0.00/0.16**	0.1/0.21
AvgTime (s)	1862.55	**1712.93**	1839.95	1867.01	-	1757.32

*Note*. In Table 11, the symbol “-” denotes the cases when the result is not reported in the literature.

**Table 12:** Comparative results of different algorithms on the MDG-a.

Instance name	IVNS	GVNS	SGVNS	FITS	NDVNS	HA-CCP
MDG-a_21	379241.00/376249.80	388647.00/387189.20	389269.00/389208.15	383164.00/382387.05	**391259.00**/390647.15	390987.00/**390660.00**
MDG-a_22	380665.00/377418.75	384487.00/384018.35	386354.00/386007.50	380817.00/380489.60	**388476.00/387718.60**	387838.00/387393.10
MDG-a_23	375697.00/374941.20	386154.00/385489.20	387267.00/387019.40	380630.00/379334.85	**389363.00/388908.60**	389071.00/388611.10
MDG-a_24	378258.00/376540.75	387095.00/386473.15	388423.00/388008.45	381783.00/380560.55	**390269.00/389655.40**	390051.00/389525.40
MDG-a_25	389242.00/384685.70	387121.00/395749.55	398108.00/397471.50	390623.00/386346.05	**399478.00/399002.40**	399215.00/398869.20
MDG-a_26	392407.00/389113.50	396756.00/400015.60	402135.00/401471.95	393222.00/392916.10	**403457.00**/402740.95	403425.00/**402759.75**
MDG-a_27	375566.00/373113.95	380243.00/379248.65	381896.00/381208.70	377617.00/376721.60	**383780.00/382951.05**	383246.00/382731.55
MDG-a_28	378486.00/375546.50	386018.00/385207.20	387594.00/387004.55	380748.00/379732.95	**389025.00/388516.50**	388553.00/388208.60
MDG-a_29	377175.00/372663.75	382108.00/381301.50	383937.00/383008.55	378239.00/377540.40	**385316.00/384816.05**	384933.00/384588.45
MDG-a_30	389615.00/386318.15	394817.00/394281.50	396678.00/396041.70	389452.00/389254.10	**398211.00/397776.80**	398004.00/397503.15
MDG-a_31	376920.00/374627.70	384718.00/384018.25	386587.00/385749.40	379407.00/378922.70	**388375.00/387562.05**	387609.00/387276.55
MDG-a_32	382405.00/379790.40	391693.00/391004.60	393098.00/392578.60	383333.00/382737.35	**394611.00/394031.35**	394290.00/393941.40
MDG-a_33	377689.00/373096.60	382576.00/381876.80	384291.00/383273.45	379047.00/376641.80	**385806.00/385212.70**	385583.00/385036.20
MDG-a_34	384412.00/381472.65	393135.00/392409.55	394676.00/394176.80	386544.00/385459.25	**396725.00/395823.10**	396214.00/395706.85
MDG-a_35	385251.00/383302.15	393384.00/392174.20	394687.00/394059.40	386347.00/385302.60	**396054.00**/395455.20	395848.00/**395472.25**
MDG-a_36	394867.00/389332.30	400271.00/400019.40	402087.00/401287.65	396368.00/394103.05	**403604.00/403111.10**	402998.00/402698.45
MDG-a_37	384811.00/380906.40	387006.00/386276.45	388411.00/387875.60	386195.00/385797.30	**390289.00/389612.25**	389769.00/389349.00
MDG-a_38	387472.00/384752.55	394264.00/393879.15	395865.00/395287.90	388712.00/387807.15	**397407.00**/396710.05	397249.00/**396775.25**
MDG-a_39	381738.00/378840.90	390008.00/389147.50	391415.00/391074.95	385975.00/383036.10	**393415.00/392799.15**	392913.00/392506.70
MDG-a_40	392751.00/391517.70	403662.00/403108.90	405184.00/405008.90	396321.00/395505.80	**407084.00/406615.75**	406672.00/406245.60
#Best	0/0	0/0	0/0	0/0	**20/16**	0/4
*p* Value	7.74*E* − 06/7.74*E* − 06	7.74*E* − 06/7.74*E* − 06	7.74*E* − 06/7.74*E* − 06	7.74*E* − 06/7.74e-06	7.74E-06/0.01	

*Note*. In Table 12, data A/B denote the best objective value and the average objective value of the instance found by the algorithm.

**Table 13:** Dev_best_, Dev_avg_, and average time of different algorithms on the MDG-a.

Instance name	IVNS	GVNS	SGVNS	FITS	NDVNS	HA-CCP
MDG-a_21	3.07/3.84/1938.05	0.67/1.04/1518.3	0.51/0.52/1867.83	2.07/2.27/1872.28	0.00/0.16/-	0.07/0.15/1765.62
MDG-a_22	2.01/2.85/1840.87	1.03/1.15/1806.64	0.55/0.64/1801.35	1.97/2.06/1931.75	0.00/0.19/-	0.16/0.28/1719.05
MDG-a_23	3.51/3.7/1868.53	0.82/0.99/1741.74	0.54/0.60/1839.26	2.24/2.58/1937.18	0.00/0.12/-	0.07/0.19/1773.65
MDG-a_24	3.08/3.52/1892.93	0.81/0.97/1697.71	0.47/0.58/1830.54	2.17/2.49/1903.31	0.00/0.16/-	0.06/0.19/1807.82
MDG-a_25	2.56/3.7/1829.42	3.09/0.93/1663.95	0.34/0.50/1870.12	2.22/3.29/1824.79	0.00/0.12/-	0.07/0.15/1731.29
MDG-a_26	2.74/3.56/1923.60	1.66/0.85/1665.89	0.33/0.49/1790.9	2.54/2.61/1926.98	0.00/0.18/-	0.01/0.17/1701.12
MDG-a_27	2.14/2.78/1790.23	0.92/1.18/1635.81	0.49/0.67/1890.33	1.61/1.84/1837.59	0.00/0.22/-	0.14/0.27/1630.26
MDG-a_28	2.71/3.46/1920.76	0.77/0.98/1681.61	0.37/0.52/1879.05	2.13/2.39/1733.96	0.00/0.13/-	0.12/0.21/1849.73
MDG-a_29	2.11/3.28/1903.37	0.83/1.04/1835.42	0.36/0.60/1859.38	1.84/2.02/1913.92	0.00/0.13/-	0.10/0.19/1780.88
MDG-a_30	2.16/2.99/1820.66	0.85/0.99/1692.74	0.38/0.54/1793.7	2.20/2.25/1867.39	0.00/0.11/-	0.05/0.18/1801.66
MDG-a_31	2.95/3.54/1912.22	0.94/1.12/1691.17	0.46/0.68/1789.28	2.31/2.43/1703.24	0.00/0.21/-	0.20/0.28/1637.96
MDG-a_32	3.09/3.76/1888.87	0.74/0.91/1663.5	0.38/0.52/1853.43	2.86/3.01/1913.13	0.00/0.15/-	0.08/0.17/1739.88
MDG-a_33	2.10/3.29/1908.54	0.84/1.02/1816.25	0.39/0.66/1908.27	1.75/2.38/1960.62	0.00/0.15/-	0.06/0.2/1788.08
MDG-a_34	3.10/3.84/1812.31	0.90/1.09/1759.61	0.52/0.64/1840.43	2.57/2.84/1820.16	0.00/0.23/-	0.13/0.26/1721.00
MDG-a_35	2.73/3.22/1810.68	0.67/0.98/1661.62	0.35/0.50/1797.99	2.45/2.71/1895.51	0.00/0.15/-	0.05/0.15/1827.95
MDG-a_36	2.16/3.54/1838.15	0.83/0.89/1700.47	0.38/0.57/1842.45	1.79/2.35/1715.11	0.00/0.12/-	0.15/0.22/1731.4
MDG-a_37	1.40/2.40/1851.54	0.84/1.03/1752.65	0.48/0.62/1833.76	1.05/1.15/1935.18	0.00/0.17/-	0.13/0.24/1746.95
MDG-a_38	2.50/3.18/1863.12	0.79/0.89/1769.98	0.39/0.53/1804.21	2.19/2.42/1909.01	0.00/0.18/-	0.04/0.16/1806.92
MDG-a_39	2.97/3.7/1901.77	0.87/1.08/1732.95	0.51/0.59/1887.26	1.89/2.64/1874.83	0.00/0.16/-	0.13/0.23/1763.7
MDG-a_40	3.52/3.82/1735.43	0.84/0.98/1770.49	0.47/0.51/1819.48	2.64/2.84/1864.35	0.00/0.12/-	0.10/0.21/1821.44
Average	2.63/3.4/1862.55	0.99/1.01/1712.93	0.43/0.57/1839.95	2.12/2.43/1867.01	0.00/0.16/-	0.1/0.21/1757.32

*Note*. In Table 13, data A/B/C denote the value of Dev_best_, the value of Dev_avg_, and the algorithm's average time to find a final solution. The symbol “-” denotes the cases when the result is not reported in the literature.

**Table 14:** Success rate of RCM and CM2 in constructing initial feasible solution on RanReal960 instances with *p*=60.

Instance name	(1.30, 1)	(1.28, 1)	(1.27, 1)	(1, 1)	(1, 0.94)	(1, 0.93)	(1, 0.92)
RanReal960_01.60	100%/100%	100%/100%	100%/100%	100%/100%	85%/100%	0%/100%	0%/100%
RanReal960_02.60	97%/100%	100%/100%	100%/100%	100%/100%	100%/100%	100%/100%	90%/100%
RanReal960_03.60	100%/100%	100%/100%	100%/100%	100%/100%	100%/100%	100%/100%	20%/100%
RanReal960_04.60	1%/14%	100%/100%	100%/100%	100%/100%	100%/100%	100%/100%	100%/100%
RanReal960_05.60	100%/100%	100%/100%	100%/100%	100%/100%	100%/100%	64%/100%	0%/100%
RanReal960_06.60	100%/100%	100%/100%	100%/100%	100%/100%	34%/100%	0%/100%	0%/0%
RanReal960_07.60	100%/100%	100%/100%	100%/100%	100%/100%	0%/100%	0%/0%	0%/0%
RanReal960_08.60	0%/0%	71%/95%	79%/98%	100%/100%	100%/100%	100%/100%	100%/100%
RanReal960_09.60	100%/100%	100%/100%	100%/100%	100%/100%	100%/100%	100%/100%	89%/100%
RanReal960_10.60	100%/100%	100%/100%	100%/100%	100%/100%	64%/100%	0%/100%	0%/100%
Average	79.8%/**81.4%**	97.1%/**99.5%**	97.9%/**99.8%**	**100%/100%**	78.3%/**100%**	56.4%/**90%**	39.9%/**80%**

*Note*. In Table 14, *s*_1_ and *s*_2_ in (*s*_1_, *s*_2_), respectively, denote the scaling ratios of the lower and upper bounds of the cluster capacity. *A* and *B* in *A*/*B,* respectively, represent the initial solution construction success rate obtained using RCM and CM2.

**Table 15:** Statistical comparison results of HA-CCP-RCM and HA-CCP.

	HA-CCP-RCM	HA-CCP
#Best/Avg	4/4	**8/8**
*p* value_best_/*p* value_avg_	0.39/0.39	
Avg Dev_best_/Dev_avg_ (%)	0.04/0.14	**0.01/0.13**
AvgTime (s)	**650.18**	659.04

**Table 16:** Comparative results of HA-CCP-RCM and HA-CCP.

Instance name	HA-CCP-RCM	HA-CCP
RanReal240_05	**195558.15/195472.31**	195540.41/195469.00
RanReal240_09	**209186.90/208970.18**	209057.64/208897.23
RanReal240_16	204063.69/203943.95	**204072.57/203961.38**
RanReal480_05	484845.80/**484337.08**	**485138.44**/484092.59
RanReal480_14	515313.22/514565.40	**515476.42/514801.49**
RanReal480_18	**526331.30**/525686.10	526314.82/**525702.47**
RanReal960_02.30	1434083.69/1432684.79	**1435087.46/1433503.63**
RanReal960_05.60	748689.45/**747833.47**	**749316.68**/747647.96
RanReal960_06.30	1419623.57/1418217.77	**1420942.81/1418222.26**
RanReal960_07.40	**1043459.88**/1041763.79	1043176.75/**1042209.24**
MDG-a_23	388753.00/388437.80	**389071.00/388611.10**
MDG-a_35	395692.00/395378.35	**395848.00/395472.25**
#Best	4/4	**8/8**
*p* Value	0.39/0.39	

*Note*. In Table 16, data A/B denote the best objective value and the average objective value of the instance found by the algorithm.

**Table 17:** Dev_best_, Dev_avg_, and average time of HA-CCP-RCM and HA-CCP.

Instance name	HA-CCP-RCM	HA-CCP
RanReal240_05	0.00/0.04/119.45	0.01/0.05/135.27
RanReal240_09	0.00/0.10/93.85	0.06/0.14/98.89
RanReal240_16	0.00/0.06/120.73	0.00/0.05/102.24
RanReal480_05	0.06/0.17/305.23	0.00/0.22/301.91
RanReal480_14	0.03/0.18/342.13	0.00/0.13/340.90
RanReal480_18	0.00/0.12/319.68	0.00/0.12/330.79
RanReal960_02.30	0.07/0.17/773.08	0.00/0.11/731.68
RanReal960_05.60	0.08/0.20/754.65	0.00/0.22/691.55
RanReal960_06.30	0.09/0.19/733.07	0.00/0.19/777.19
RanReal960_07.40	0.00/0.16/736.56	0.03/0.12/796.50
MDG-a_23	0.08/0.16/1766.01	0.00/0.12/1773.65
MDG-a_35	0.04/0.12/1737.76	0.00/0.09/1827.95
Average	0.04/0.14/**650.18**	**0.01**/**0.13**/659.04

*Note*. In Table 17, data A/B/C denote the value of Dev_best_, the value of Dev_avg_, and the algorithm's average time to find a final solution.

**Table 18:** Statistical comparison results of DM2 and DM3 on RanReal960.

	DM2	DM3
#Best/Avg	1/0	**29/30**
*p* value_best_/*p* value_avg_	5.77*E* − 08/1.86*E* − 09	
Avg Dev_best_/Dev_avg_ (%)	0.19/0.33	**0.02/0.17**
AvgTime (s)	**697.94**	705.36

**Table 19:** Comparative results of DM2 and DM3 on RanReal960.

Instance name	DM2	DM3
RanReal960_01.30	1339422.76/1337439.23	**1339783.24/1338150.82**
RanReal960_02.30	1433976.41/1432620.51	**1434814.15/1433364.47**
RanReal960_03.30	1397391.88/1395414.88	**1397392.10/1395893.73**
RanReal960_04.30	1413805.22/1411624.30	**1414298.90/1412090.36**
RanReal960_05.30	1369728.30/1368811.02	**1370975.25/1369468.75**
RanReal960_06.30	**1419316.66**/1417731.23	1418984.70/**1417747.24**
RanReal960_07.30	1340663.25/1339585.27	**1340800.86/1339709.55**
RanReal960_08.30	1466312.51/1465036.05	**1467896.40/1465355.37**
RanReal960_09.30	1385370.30/1383667.64	**1386030.28/1384583.74**
RanReal960_10.30	1384748.37/1383091.10	**1385179.70/1383692.23**
RanReal960_01.40	1041414.82/1040042.65	**1042465.91/1041298.30**
RanReal960_02.40	1115375.82/1113835.01	**1117118.83/1115382.72**
RanReal960_03.40	1087242.79/1086124.89	**1089455.26/1087348.95**
RanReal960_04.40	1100870.73/1099218.54	**1101967.82/1100620.80**
RanReal960_05.40	1065983.91/1064140.38	**1066146.84/1064752.74**
RanReal960_06.40	1104907.29/1103838.17	**1106596.78/1105105.35**
RanReal960_07.40	1041902.02/1040549.03	**1043198.47/1041876.18**
RanReal960_08.40	1143170.88/1141692.09	**1144718.73/1142826.31**
RanReal960_09.40	1075078.06/1073881.61	**1076824.82/1075395.86**
RanReal960_10.40	1077549.54/1076250.33	**1078910.90/1077636.68**
RanReal960_01.60	731791.05/730574.72	**734308.41/732725.73**
RanReal960_02.60	775695.34/773599.78	**778535.60/776393.83**
RanReal960_03.60	758983.07/757547.47	**761525.30/760142.69**
RanReal960_04.60	766615.64/765530.39	**770670.09/768853.66**
RanReal960_05.60	746350.92/745399.89	**749120.37/747789.87**
RanReal960_06.60	766117.84/765444.75	**768842.79/767568.44**
RanReal960_07.60	728688.71/727719.25	**732517.17/730460.65**
RanReal960_08.60	793341.85/790820.80	**795271.66/793741.94**
RanReal960_09.60	752529.87/751033.08	**754767.54/753712.19**
RanReal960_10.60	753818.27/752479.66	**755877.96/754823.36**
#Best	1/0	**29/30**
*p* value	5.77*E* − 08/1.86*E* − 09	

*Note*. In Table 19, data A/B denote the best objective value and the average objective value of the instance found by the algorithm.

**Table 20:** Dev_best_, Dev_avg_, and average time of DM2 and DM3 on RanReal960.

Instance name	DM2	DM3
RanReal960_01.30	0.04/0.18/657.10	0.01/0.13/701.09
RanReal960_02.30	0.08/0.17/764.82	0.02/0.12/797.04
RanReal960_03.30	0.01/0.15/710.61	0.01/0.12/720.84
RanReal960_04.30	0.03/0.19/763.00	0.00/0.16/667.80
RanReal960_05.30	0.12/0.19/798.16	0.03/0.14/676.75
RanReal960_06.30	0.11/0.23/714.99	0.14/0.22/647.17
RanReal960_07.30	0.07/0.15/745.90	0.06/0.14/749.34
RanReal960_08.30	0.11/0.19/754.81	0.00/0.17/676.06
RanReal960_09.30	0.05/0.17/725.25	0.00/0.10/721.36
RanReal960_10.30	0.03/0.15/645.77	0.00/0.11/725.62
RanReal960_01.40	0.10/0.23/636.56	0.00/0.11/669.23
RanReal960_02.40	0.19/0.33/662.33	0.04/0.19/678.43
RanReal960_03.40	0.20/0.31/730.12	0.00/0.19/748.76
RanReal960_04.40	0.10/0.25/658.97	0.00/0.12/732.29
RanReal960_05.40	0.02/0.19/707.52	0.00/0.13/701.30
RanReal960_06.40	0.23/0.33/703.43	0.08/0.21/654.20
RanReal960_07.40	0.12/0.25/667.78	0.00/0.13/684.48
RanReal960_08.40	0.14/0.26/702.86	0.00/0.17/689.27
RanReal960_09.40	0.16/0.27/719.46	0.00/0.13/719.93
RanReal960_10.40	0.13/0.25/717.19	0.00/0.12/706.70
RanReal960_01.60	0.34/0.51/693.50	0.00/0.22/732.69
RanReal960_02.60	0.36/0.63/734.58	0.00/0.28/718.58
RanReal960_03.60	0.33/0.52/710.39	0.00/0.18/761.03
RanReal960_04.60	0.53/0.67/707.52	0.00/0.24/707.68
RanReal960_05.60	0.40/0.52/632.37	0.03/0.20/666.28
RanReal960_06.60	0.41/0.49/607.12	0.05/0.22/667.62
RanReal960_07.60	0.52/0.65/701.64	0.00/0.28/642.39
RanReal960_08.60	0.25/0.56/604.62	0.01/0.20/707.20
RanReal960_09.60	0.30/0.50/621.11	0.01/0.15/747.19
RanReal960_10.60	0.29/0.47/738.65	0.02/0.16/742.39
Average	0.19/0.33/**697.94**	**0.02**/**0.17**/705.36

*Note*. In Table 20, data A/B/C denote the value of Dev_best_, the value of Dev_avg_, and the algorithm's average time to find a final solution.

**Table 21:** Statistical comparison results of HA-CCP-RS and HA-CCP on the MDG-a.

	HA-CCP-RS	HA-CCP
#Best/Avg	0/0	**20/20**
*p* value_*best*_/*p* value_avg_	1.91*E* − 06/1.91*E* − 06	
Avg Dev_best_/Dev_avg_ (%)	0.12/0.24	**0.00/0.11**
AvgTime (s)	1843.11	**1757.32**

**Table 22:** Comparative results of HA-CCP-RS and HA-CCP on the MDG-a.

Instance name	HA-CCP-RS	HA-CCP
MDG-a_21	390621.00/390145.80	**390987.00/390660.00**
MDG-a_22	387550.00/387010.65	**387838.00/387393.10**
MDG-a_23	388466.00/387984.40	**389071.00/388611.10**
MDG-a_24	389555.00/389016.65	**390051.00/389525.40**
MDG-a_25	398779.00/398257.00	**399215.00/398869.20**
MDG-a_26	402852.00/402381.40	**403425.00/402759.75**
MDG-a_27	382574.00/382245.40	**383246.00/382731.55**
MDG-a_28	388264.00/387758.55	**388553.00/388208.60**
MDG-a_29	384292.00/383914.05	**384933.00/384588.45**
MDG-a_30	397474.00/397111.05	**398004.00/397503.15**
MDG-a_31	387298.00/386716.30	**387609.00/387276.55**
MDG-a_32	393730.00/393350.75	**394290.00/393941.40**
MDG-a_33	385030.00/384564.15	**385583.00/385036.20**
MDG-a_34	395459.00/394970.90	**396214.00/395706.85**
MDG-a_35	395459.00/394970.90	**395848.00/395472.25**
MDG-a_36	402659.00/402316.25	**402998.00/402698.45**
MDG-a_37	389236.00/388922.00	**389769.00/389349.00**
MDG-a_38	396689.00/396276.55	**397249.00/396775.25**
MDG-a_39	392441.00/392042.25	**392913.00/392506.70**
MDG-a_40	406367.00/405899.70	**406672.00/406245.60**
#Best	0/0	**20/20**
*p* value	1.91*E* − 06/1.91*E* − 06	

*Note*. In Table 22, data A/B denote the best objective value and the average objective value of the instance found by the algorithm.

**Table 23:** Dev_best_, Dev_avg_, and average time of HA-CCP-RS and HA-CCP on the MDG-a.

Instance name	HA-CCP-RS	HA-CCP
MDG-a_21	0.09/0.22/1827.80	0.00/0.08/1765.62
MDG-a_22	0.07/0.21/1854.63	0.00/0.11/1719.05
MDG-a_23	0.16/0.28/1760.08	0.00/0.12/1773.65
MDG-a_24	0.13/0.27/1876.73	0.00/0.13/1807.82
MDG-a_25	0.11/0.24/1854.49	0.00/0.09/1731.29
MDG-a_26	0.14/0.26/1854.90	0.00/0.16/1701.12
MDG-a_27	0.18/0.26/1770.48	0.00/0.13/1630.26
MDG-a_28	0.07/0.20/1842.33	0.00/0.09/1849.73
MDG-a_29	0.17/0.26/1856.95	0.00/0.09/1780.88
MDG-a_30	0.13/0.22/1888.56	0.00/0.13/1801.66
MDG-a_31	0.08/0.23/1833.46	0.00/0.09/1637.96
MDG-a_32	0.14/0.24/1811.64	0.00/0.09/1739.88
MDG-a_33	0.14/0.26/1827.21	0.00/0.14/1788.08
MDG-a_34	0.19/0.31/1822.35	0.00/0.13/1721.00
MDG-a_35	0.10/0.22/1822.35	0.00/0.09/1827.95
MDG-a_36	0.08/0.17/1839.01	0.00/0.07/1731.40
MDG-a_37	0.14/0.22/1849.99	0.00/0.11/1746.95
MDG-a_38	0.14/0.24/1904.83	0.00/0.12/1806.92
MDG-a_39	0.12/0.22/1894.74	0.00/0.10/1763.70
MDG-a_40	0.07/0.19/1869.64	0.00/0.10/1821.44
Average	0.12/0.24/1843.11	**0.00/0.11/1757.32**

*Note*. In Table 23, data A/B/C denote the value of Dev_best_, the value of Dev_avg_, and the algorithm's average time to find a final solution.

**Table 24:** Statistical comparison results of HA-CCP-NAC and HA-CCP on RanReal240 and RanReal480.

	HA-CCP-NAC	HA-CCP
#Best/Avg	0/0	**40/40**
*p* value_best_/*p* value_avg_	1.82*E* − 12/1.82*E* − 12	
Avg Dev_best_/Dev_avg_ (%)	0.17/0.26	**0.00/0.11**
AvgTime (s)	**190.29**	217.25

**Table 25:** Comparative results of HA-CCP-NAC and HA-CCP on RanReal240 and RanReal480.

Instance name	HA-CCP-NAC	HA-CCP
RanReal240_01	224727.06/224616.93	**225003.70/224897.01**
RanReal240_02	204435.05/204256.39	**204624.36/204515.06**
RanReal240_03	198819.54/198592.41	**199079.37/198915.84**
RanReal240_04	225102.36/224814.97	**225683.17/225346.54**
RanReal240_05	195307.90/195203.22	**195540.41/195469.00**
RanReal240_06	216477.84/216312.91	**216730.26/216613.93**
RanReal240_07	208949.52/208789.41	**209282.88/209150.41**
RanReal240_08	205040.86/204835.75	**205246.82/205110.51**
RanReal240_09	208588.26/208475.27	**209057.64/208897.23**
RanReal240_10	192868.91/192706.85	**193044.16/192954.72**
RanReal240_11	204549.74/204455.01	**204722.75/204638.07**
RanReal240_12	200951.37/200791.23	**201117.11/201006.45**
RanReal240_13	202170.78/201988.4	**202345.48/202285.83**
RanReal240_14	228594.33/228434.38	**228971.03/228740.72**
RanReal240_15	191160.90/190978.45	**191243.76/191117.60**
RanReal240_16	203796.06/203691.26	**204072.57/203961.38**
RanReal240_17	194915.27/194730.53	**195393.97/195278.93**
RanReal240_18	195012.27/194868.93	**195167.14/195069.80**
RanReal240_19	199135.59/198980.50	**199307.33/199204.90**
RanReal240_20	211956.00/211831.11	**212229.46/212111.19**
RanReal480_01	555335.92/554734.67	**556126.86/555338.06**
RanReal480_02	510361.31/509822.51	**511566.55/510924.27**
RanReal480_03	496848.36/496025.80	**498028.54/497109.59**
RanReal480_04	521736.02/521332.69	**522790.22/521999.31**
RanReal480_05	483933.52/483612.70	**485138.44/484092.59**
RanReal480_06	533914.73/533474.70	**534961.79/534114.47**
RanReal480_07	545679.18/545247.24	**546503.90/545582.87**
RanReal480_08	531790.95/531213.90	**532891.23/532161.08**
RanReal480_09	555943.11/555557.53	**557120.25/556265.26**
RanReal480_10	519486.75/519083.16	**520492.06/519891.18**
RanReal480_11	523048.09/522469.89	**524669.99/523760.48**
RanReal480_12	501724.17/500933.49	**503359.96/502069.60**
RanReal480_13	534164.65/533751.60	**535251.73/534707.51**
RanReal480_14	513914.91/513549.56	**515476.42/514801.49**
RanReal480_15	517055.94/516347.68	**518370.90/517283.29**
RanReal480_16	549618.65/549232.56	**550317.71/549881.85**
RanReal480_17	537155.63/536696.66	**538745.70/537826.26**
RanReal480_18	525517.30/524655.46	**526314.82/525702.47**
RanReal480_19	522191.15/521731.11	**522958.23/522365.10**
RanReal480_20	518068.63/517724.40	**519277.92/518518.27**
#Best	0/0	**40/40**
*p* value	1.82*E* − 12/1.82*E* − 12	

*Note*. In Table 25, data A/B denote the best objective value and the average objective value of the instance found by the algorithm.

**Table 26:** Dev_best_, Dev_avg_, and average time of HA-CCP-NAC and HA-CCP on RanReal240 and RanReal480.

Instance name	HA-CCP-NAC	HA-CCP
RanReal240_01	0.12/0.17/93.82	0.00/0.05/127.88
RanReal240_02	0.09/0.18/127.36	0.00/0.05/111.58
RanReal240_03	0.13/0.24/130.94	0.00/0.08/135.66
RanReal240_04	0.26/0.38/122.88	0.00/0.15/124.67
RanReal240_05	0.12/0.17/110.23	0.00/0.04/135.27
RanReal240_06	0.12/0.19/109.10	0.00/0.05/129.28
RanReal240_07	0.16/0.24/123.43	0.00/0.06/127.25
RanReal240_08	0.10/0.20/100.10	0.00/0.07/112.98
RanReal240_09	0.22/0.28/114.32	0.00/0.08/98.89
RanReal240_10	0.09/0.17/116.08	0.00/0.05/111.58
RanReal240_11	0.08/0.13/122.57	0.00/0.04/125.15
RanReal240_12	0.08/0.16/131.99	0.00/0.06/108.32
RanReal240_13	0.09/0.18/135.13	0.00/0.03/139.97
RanReal240_14	0.16/0.23/129.75	0.00/0.1/122.04
RanReal240_15	0.04/0.14/118.75	0.00/0.07/101.57
RanReal240_16	0.14/0.19/145.53	0.00/0.05/102.24
RanReal240_17	0.24/0.34/132.89	0.00/0.06/132.10
RanReal240_18	0.08/0.15/122.70	0.00/0.05/110.87
RanReal240_19	0.09/0.16/80.49	0.00/0.05/121.71
RanReal240_20	0.13/0.19/103.68	0.00/0.06/99.55
RanReal480_01	0.14/0.25/197.77	0.00/0.14/349.71
RanReal480_02	0.24/0.34/284.81	0.00/0.13/330.24
RanReal480_03	0.24/0.40/295.48	0.00/0.18/332.28
RanReal480_04	0.20/0.28/267.30	0.00/0.15/329.59
RanReal480_05	0.25/0.31/258.95	0.00/0.22/301.91
RanReal480_06	0.20/0.28/251.78	0.00/0.16/280.00
RanReal480_07	0.15/0.23/303.63	0.00/0.17/285.02
RanReal480_08	0.21/0.31/275.77	0.00/0.14/283.23
RanReal480_09	0.21/0.28/208.49	0.00/0.15/255.35
RanReal480_10	0.19/0.27/257.74	0.00/0.12/307.38
RanReal480_11	0.31/0.42/214.08	0.00/0.17/299.74
RanReal480_12	0.32/0.48/277.58	0.00/0.26/349.86
RanReal480_13	0.20/0.28/271.16	0.00/0.10/333.59
RanReal480_14	0.30/0.37/293.64	0.00/0.13/340.90
RanReal480_15	0.25/0.39/279.55	0.00/0.21/282.23
RanReal480_16	0.13/0.20/243.76	0.00/0.08/294.16
RanReal480_17	0.30/0.38/251.44	0.00/0.17/336.43
RanReal480_18	0.15/0.32/297.75	0.00/0.12/330.79
RanReal480_19	0.15/0.23/268.87	0.00/0.11/343.32
RanReal480_20	0.23/0.30/240.17	0.00/0.15/345.87
Average	0.17/0.26/**190.29**	**0.00/0.11**/217.25

*Note*. In Table 26, data A/B/C denote the value of Dev_best_, the value of Dev_avg_, and the algorithm's average time to find a final solution.

## Data Availability

The data are available at CCPLIB (https://grafo.etsii.urjc.es/optsicom/ccp/ccplib.zip).
